# Slx5-Slx8 ubiquitin ligase targets active pools of the Yen1 nuclease to limit crossover formation

**DOI:** 10.1038/s41467-018-07364-x

**Published:** 2018-11-27

**Authors:** Ibtissam Talhaoui, Manuel Bernal, Janet R. Mullen, Hugo Dorison, Benoit Palancade, Steven J. Brill, Gerard Mazón

**Affiliations:** 1 CNRS UMR 8200, Université Paris-Sud - Université Paris-Saclay, Gustave Roussy, 114 rue Édouard Vaillant, 94800 Villejuif, France; 2 Department of Molecular Biology and Biochemistry, Rutgers University, Piscataway, NJ 08854 USA; 3 Institut Jacques Monod, CNRS UMR 7592, Université Paris Diderot, Sorbonne Paris Cité, 15 rue Hélène Brion, 75013 Paris, France

## Abstract

The repair of double-stranded DNA breaks (DSBs) by homologous recombination involves the formation of branched intermediates that can lead to crossovers following nucleolytic resolution. The nucleases Mus81-Mms4 and Yen1 are tightly controlled during the cell cycle to limit the extent of crossover formation and preserve genome integrity. Here we show that Yen1 is further regulated by sumoylation and ubiquitination. In vivo, Yen1 becomes sumoylated under conditions of DNA damage by the redundant activities of Siz1 and Siz2 SUMO ligases. Yen1 is also a substrate of the Slx5-Slx8 ubiquitin ligase. Loss of Slx5-Slx8 stabilizes the sumoylated fraction, attenuates Yen1 degradation at the G1/S transition, and results in persistent localization of Yen1 in nuclear foci. Slx5-Slx8-dependent ubiquitination of Yen1 occurs mainly at K714 and mutation of this lysine increases crossover formation during DSB repair and suppresses chromosome segregation defects in a *mus81∆* background.

## Abstract (web summary)

Nucleases are regulated during the cell cycle to control for crossover formation and maintain genome integrity. Here the authors reveal that the yeast Holliday junction resolvase Yen is a sumoylation target and it is regulated by the ubiquitin ligases Slx5/Slx8 during crossover formation.

## Introduction

Homologous recombination (HR) is a key repair pathway for the maintenance of genome integrity. HR is involved in the repair of double-strand breaks (DSB) generated by endogenous or exogenous sources of DNA damage and it plays an important role in the repair of damage associated with DNA replication^1^. A variety of DNA joint molecules (JM) form during the different steps of the HR pathway and these are sequentially matured into novel intermediates or dismantled by different specialized proteins to prevent their persistence into mitosis. Failure to resolve joint-molecule intermediates results in chromosome segregation defects^1–4^. In yeast, the helicase Sgs1, together with Rmi1 and Top3, mediates the dissolution of double Holliday Junctions (dHJ) to ensure a non-crossover outcome^2,3^, and similar NCO outcomes are generated by helicases, such as Mph1 or Srs2^4–6^. In contrast to the dissolution pathways, nucleolytic processing of recombination intermediates can result in reciprocal crossovers (CO), with the risk of loss of heterozygosity (LOH), or chromosome translocations, both of which are genome-destabilizing events^1,7^.

Nucleolytic processing of HR intermediates is strictly controlled and appears to be used as a last option to cope with orphan HJs and other intermediates that cannot be dissolved by the Sgs1-mediated pathway^4,8^. Whereas Mus81-Mms4 is hyper activated in late G2/M phase by Cdc5- and Cdc28/CDK1-dependent phosphorylation of Mms4^9–11^, Cdc28 phosphorylates Yen1 to prevent its activity and nuclear localization until anaphase^12,13^. In anaphase, the Cdc14 phosphatase gradually dephosphorylates Yen1, and this late activation of Yen1 ensures that persistent recombination intermediates are resolved before mitotic exit^12,13^. Although CO levels are minimized by the late activation of nucleases, their windows of activity are likely to overlap with those of DNA helicases that dissociate intermediates to form NCOs. It is thus possible that another layer of control is required subsequent to chromatin binding to prevent the use of nucleases when other factors are available. The tight regulation of these nucleases also highlights the risk of their uncontrolled activity in other cell-cycle phases, and suggests that their turnover might be enforced to remove active pools from the nucleus when they are no longer needed.

Regulation by coupling of the small ubiquitin-like modifier (SUMO)^14^ has emerged as a potent means to fine tune the amount and activity of specific pools of proteins, especially during DNA-mediated transactions^15^. In *Saccharomyces cerevisiae*, the enzymes involved in SUMO conjugation are the E1 Aos1-Uba2 activating enzyme dimer, the E2 conjugating enzyme Ubc9, and a limited set of E3 ligases (including Siz1, Siz2, and Mms21) that provide substrate selectivity^16–18^. Although protein sumoylation regulates multiple cellular activities, it has been shown to be especially important during the DNA damage response^19–21^. Important players of the HR pathway are found among the sumoylated DNA repair targets, including Rad52, PCNA, RPA, and Sgs1^20,22–26^. Some lines of evidence link sumoylation to specific pathways that locally target repair factors to degradation by the action of SUMO-targeted ubiquitin ligases (STUbLs)^27,28^ to prevent the toxic effects of their persistent activation. Two STUbLs are thought to operate in *S.cerevisiae*, the Slx5–Slx8 complex^29,30^ and the Uls1 protein^31,32^. Mutations in *SLX5* and *SLX8* result in slow growth or lethality in combination with components of the SUMO metabolic pathway^33^ highlighting its role in regulating sumoylated proteins. The *SLX5* and *SLX8* genes were originally identified by their requirement for the viability of *sgs1*∆ cells^34^, and this lethality is partially explained by the accumulation of sumoylated substrates in the *sgs1*∆ background.

In this work, we explored the hypothesis that there is crosstalk between Slx5–Slx8 and Yen1. We demonstrate that Yen1 is a sumoylation target and that Slx5–Slx8 participates in its regulation by ubiquitination of its lysine 714. Slx5–Slx8 prevents persistent accumulation of a fraction of Yen1 associated with sites of activity in late G2/M and helps maintain the balance between pro- and anti-crossover pathways during HR.

## Results

### Yen1 is sumoylated after DNA damage via Siz1/Siz2

STUbLs have been shown to recognize sumoylated substrates^27^, including certain nuclear proteins that participate in the DNA damage response^28^. Because Yen1 was identified in a screen for sumoylated proteins following DNA damage^25^, we analyzed how Yen1 is sumoylated in vivo and whether it interacts with Slx5–Slx8.

**Fig. 1:**
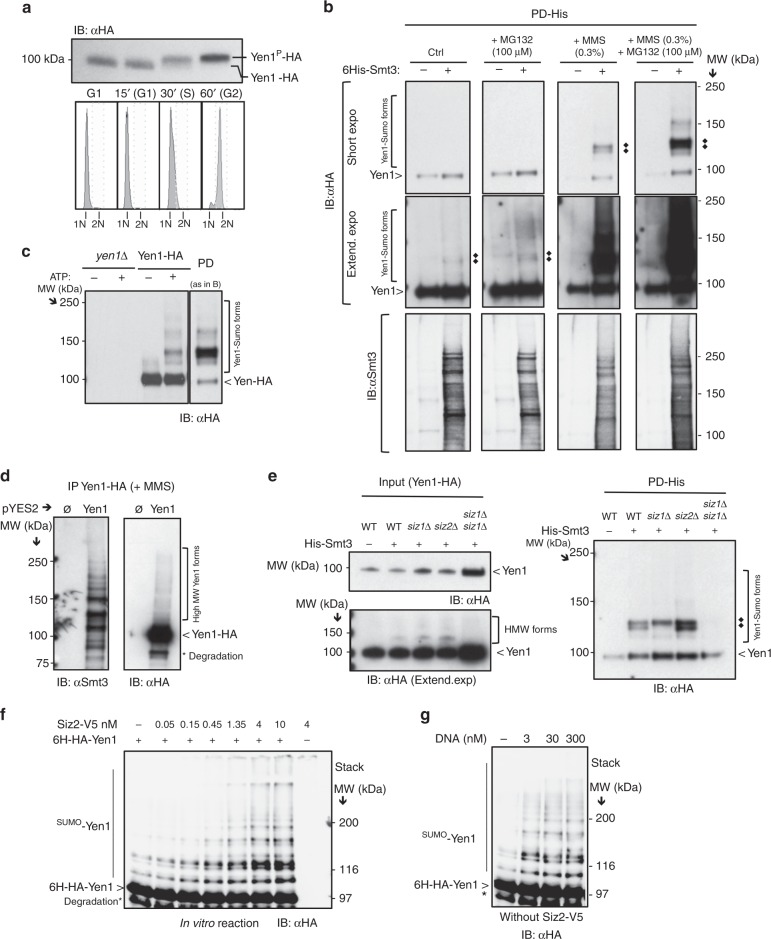
Yen1 is sumoylated in vivo and in vitro. **a** A wild-type chromosomally tagged *YEN1-HA* strain was synchronized with alpha factor and released into fresh medium to observe phosphorylation of Yen1 by immunoblot (upper) and progression through the cell cycle by FACS (lower). **b** Wild-type strains expressing Yen1-HA, with (+) or without (−) pCUP-6xHIS-Smt3, were subjected to MMS challenge followed by denaturing Ni-NTA pull-down and immunoblot analysis. Yen1 was detected by anti-HA (top and middle) and a prominent sumoylated doublet is indicated (black rhombus). Membranes were also probed with anti-Smt3 (bottom). Note that un-sumoylated Yen1 binds to Ni due to a histine-rich region. **c** Yen1-HA was overexpressed in wild-type asynchronous cells, immunoprecipitated with anti-HA, eluted by HA peptide competition and mixed with Aos1-Uba2, Ubc9, and Smt3-3KR in the presence or absence of ATP. After immunoblotting with anti-HA sumoylated forms were detected in the presence of ATP that migrate at similar sizes to those detected in the PD experiments shown in **b** (far right duplicate for comparison). A control reaction was made with HA-immunoprecipitation of a *yen1*∆ strain eluted with the same amount of HA peptide. **d** *yen1*∆ cells expressing Yen1-HA from a Gal-inducible plasmid or harboring a control plasmid were subjected to MMS treatment (0.03%), and extracts were immunoprecipitated using anti-HA prior to immunoblotting with anti-Smt3 (left) or anti-HA (right). **e** Indicated strains (WT, *siz1∆, siz2∆*, or *siz1∆ siz2∆*) with (+) or without (−) pCUP-6xHIS-Smt3 were subjected to pull-down analysis of Smt3 as in **b** in conditions of MMS damage and eluates were analysed by immunoblot. The input used for PD was immunoblotted to allow normalization and comparison between strains (bottom). **f** Purified recombinant 6His-HA-Yen1 protein was incubated under sumoylation conditions with the indicated concentrations of Siz2 followed by immunoblotting with anti-HA. Asterisk indicates breakdown products of Yen1 carried from purification. **g** Sumoylation reactions were performed as in **f** but with increasing amounts of synthetic Holliday junction DNA, 458 nM Yen1 and in the absence of Siz2. All experiments were independently replicated at least three times and images are representative of the reproducible results obtained

To detect the Yen1 post-translational modification of Yen1 by SUMO and ubiquitin (Ub), we tagged the endogenous gene with a single C–terminal HA epitope. The *YEN1-HA* allele was judged to be functional as it showed no effect on the methyl-methane sulfonate (MMS) sensitivity of a *mus81*∆ strain (Supplementary Figure 1a). Further, Yen1-HA immunoblots revealed phosphorylated and unphosphorylated forms with the expected cell-cycle regulation^12^ (Fig. 1a).

Using a 6xHIS-Smt3-expressing plasmid^35^, we performed a Histidine pull-down experiment to detect Yen1 among the sumoylated proteins. Sumoylated Yen1 was detected as a faint signal in the eluates of unperturbed cells (Fig. 1b, extended exposure) and the recovery of sumoylated Yen1 was not greatly increased by inhibiting proteasomal degradation by treatment with MG132 (Fig. 1b). However, after MMS treatment, we recovered a clear ladder of sumoylated Yen1 forms whose abundance increased in the presence of MG132 (Fig. 1b).

The Yen1-SUMO conjugates migrated as a doublet of two discrete bands near 120 KDa (Fig. 1b), these forms shifted when using a different tagged version of Smt3 further confirming their sumoylated nature (Supplementary Figure 1). We also observed higher MW bands may be due to multiple sumoylations or chains of SUMO or Ub. The major bands detected from pull-downs resemble those detected following in vitro sumoylation assays where immunoprecipitated Yen1-HA was incubated with purified Smt3-3KR, E1 (Aos1-Uba2), and E2 (Ubc9) enzymes (Fig. 1c, Supplementary Figure 1c). Sumoylation under mild MMS treatment was also detected with Yen1-HA immunoprecipitation after lysis in denaturing conditions of cells with induced expression of Yen1-HA but endogenous levels of Smt3 (Fig. 1d). We did not detect significant Yen1 sumoylation in pull-downs from *siz1∆ siz2∆* double-mutant strains confirming the requirement of these E3 ligases in vivo. Interestingly, one of the two major Yen1 sumoylation bands disappeared in the *siz1*∆ single-mutant pull-down, suggesting some sites have a strong preference for Siz1 as E3 ligase (Fig. 1e).

To complement our in vivo observations, we tested whether highly purified Yen1 produced in *E. coli* was a substrate for sumoylation in vitro. A sumoylation reaction consisting of Smt3, Aos1-Uba2 (E1), Ubc9 (E2), and Siz2 (E3), triggered the formation of Yen1 products that migrated as a ladder of bands with a more intense band at 120 KDa similar to the major forms observed in vivo (Fig. 1f). Increased concentrations of Siz2 stimulated Yen1 sumoylation, which occurred at a lower yield in the absence of E3 (Fig. 1f, g). Similar to what has already been reported for other proteins^36,37^, we observed increased Yen1 sumoylation in vitro in the presence of its DNA substrate (Fig. 1g). We conclude that the Yen1 forms detected following in vitro sumoylation are largely reminiscent of those detected in vivo (Fig. 1b). The fact that Yen1 sumoylation is stimulated by MMS treatment suggest that it is a response to substrates that accumulate during DNA damage.

To test whether substrate recognition is important for sumoylation of Yen1 in vivo, we introduced amino acid substitutions in the four central CDK1 sites to generate phospho-deleted or phospho-mimic alleles. Previous work has shown that phospho-mimic mutations in the central CDK1 sites of Yen1 impair substrate cleavage and reduce its association with DNA^12,13^. However, analysis of Smt3 pull-downs following MMS treatment showed no significant difference in sumoylation compared to the wild-type Yen1 (Supplementary Figure 1d). To directly test the possibility that the Yen1 sumoylation is stimulated by its substrates, we analysed Smt3 pull-downs from a *dna2∆ pif1* strain. It has been shown that Yen1 is important in eliminating replication-dependent recombination intermediates in this strain^38^. Compared to wild-type, more abundant sumoylation was observed in *dna2∆ pif1* cells (Supplementary Figure 1d). This supports the idea that sumoylation of Yen1 occurs in a context where Yen1 activity is required.

### Yen1 is a substrate of the Slx5–Slx8 ubiquitin ligase

**Fig. 2:**
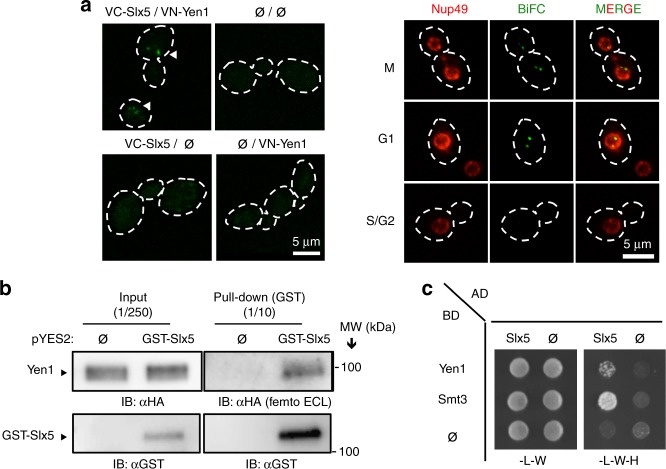
Yen1 interacts with Slx5–Slx8 in the nucleus. **a** Diploid strains carrying one allele of galactose inducible VC-Slx5 and VN-Yen1 with wild-type copies of *YEN1* and *SLX5* in the homologous chromosomes were observed by live microscopy. BiFC (white arrows) signal denotes an interaction between the two BiFC (Venus) epitopes. Control diploids lacking one or both of the epitope-tagged proteins (ϕ) were used to substract background signal. A plasmid carrying Nup49-mCherry was transformed on the diploid strain harboring VC-Slx5/VN-Yen1 to visualize the nuclear perimeter. BiFC interactions were only detected in the nuclear compartment. **b** Cells carrying either an empty vector or a pYES2 plasmid expressing GST-Slx5 under galactose control were grown in selective media and induced with galactose for 3 h. Lysates were then applied to a glutathione-sepharose column. After washing, the bound proteins were eluted and immunoblotted with α-HA (upper) or α-GST (lower). **c** Two-hybrid assays were performed with strains carrying the indicated activating domain (AD) or DNA binding domain (BD) fusions. Strains were grown in selective media lacking leucine (L) and tryptophan (W) prior to spotting on media lacking histidine (H) to detect a positive interaction. Experiments were independently replicated three times and images are representative of the reproducible results obtained

Having established that Yen1 is sumoylated, we next investigated whether the Slx5–Slx8 STUbL recognized and further processed the modified protein. To determine whether Yen1 and Slx5 physically interact or are in proximity in the nucleus, we used the bimolecular fluorescence complementation (BiFC) approach^39^. Cells expressing the complementary VN-Yen1 and VC-Slx5 epitope-tagged proteins displayed fluorescent signal in discrete foci (Fig. 2a), similar to what has been described for another Slx5–Slx8-substrate interaction^40^. The BiFC interaction was nuclear as determined by introducing a Nup49-mCherry marker (Fig. 2a). The Yen1–Slx5 interaction was confirmed using a pull-down approach. Cells containing the *YEN1-HA* allele were transformed with a pYES2 plasmid expressing GST-Slx5 under a galactose inducible promoter or an empty vector as control. After induction of GST-Slx5, cell extracts were applied to a glutathione column and Yen1 was specifically detected in the eluates (Fig. 2b). Finally, the association of Slx5 with Yen1 was further confirmed by a two-hybrid assay where DBD-Yen1 bound AD-Slx5 (Fig. 2c). These data indicate that Yen1 and Slx5 are in close proximity and may physically interact in yeast.

**Fig. 3:**
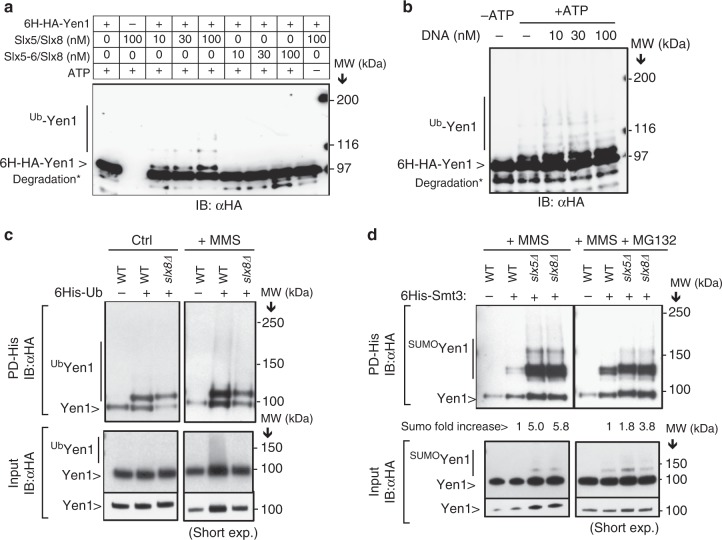
Yen1 is a direct substrate of the Slx5–Slx8 ubiquitin ligase. **a** H6-HA-Yen1 (916 nM) was ubiquitinated in vitro in the presence of the indicated concentrations of either Slx5/Slx8 or the RING mutant Slx5–6/Slx8 and 0.2 µM DNA. Control lanes with H6-HA-Yen1 in the absence of E3 and Slx5/8 in the absence of Yen1 are shown. Breakdown products of Yen1 are marked with an asterisk. **b** The ubiquitination reaction was performed as above but with 50 nM Slx5–Slx8 and increasing amounts of DNA. **c** Strains expressing 6xHis-Ub were subjected to different growth conditions and lysed to pull-down ubiquitinated proteins under denaturing conditions, input Yen1-HA levels were controlled to allow comparisons. **d** Smt3 denaturing pull-downs were performed in wild type, *slx5*∆, or *slx8*∆ cells (all in a *pdr5*∆ background) after growth in the presence of MMS. The fold increase in the sumoylated fraction indicated at the bottom of the gel is an average of three trials. Inputs were controlled in each trial to allow comparison of the eluted sumoylated proteins

The fact that Yen1 is sumoylated and interacts with the STUbL component Slx5 prompted us to investigate whether it was ubiquitinated by Slx5–Slx8. We first asked whether Slx5–Slx8 could directly recognize Yen1 as substrate by reconstituting the ubiquitination reaction in vitro. Combined with the E1 (Uba1) and E2 (Ubc5), Slx5–Slx8 ubiquitinated Yen1 producing a major band around 110 KDa and a less intense ladder of higher MW bands (Fig. 3a). No ubiquitination was detected when using a RING mutant of Slx5 in the reaction (*slx5–6*), and no cross-reacting products were detected by the anti-HA antibody when Yen1 was not added to the reaction, further confirming the specificity of detection of the Yen1 ubiquitinated forms (Fig. 3a). Ubiquitination was stimulated by the presence of DNA (Fig. 3b), suggesting that DNA enhances ubiquitination but is not essential for the reaction. Although Slx5–Slx8 is able to ubiquitinate Yen1 in vitro in the absence of prior sumoylation we cannot exclude the possibility that sumoylation is required for ubiquitination in vivo.

To determine whether the in vivo ubiquitinated Yen1 was dependent on Slx5/Slx8, we employed a pull-down approach. Using a 6xHIS-Ub expression plasmid we detected mono-ubiquitinated Yen1 in the pull-down eluates and a faint ladder of poly-ubiquitination (Fig. 3c). Yen1 ubiquitination was not largely increased under MMS treatment of cells and was not importantly altered after *slx8*∆ deletion (Fig. 3c). From the results obtained it is difficult to estimate the contribution of Slx5/8 to the Yen1 ubiquitination in vivo that also possibly involves another general turnover pathway that mask the modification of a small fraction of Yen1 by Slx5/8 as already observed for other substrates^41^.

We then explored the possibility that Slx5–Slx8-mediated degradation of Yen1 would target the sumoylated version of this protein. To this aim, we asked whether inactivation of Slx5–Slx8 affected the stability of sumoylated Yen1 detected after MMS treatment. Wild-type and cells harboring deletions of either *SLX8* or *SLX5* expressing 6xHIS-Smt3 were collected after exposure to MMS 0.3%. After Smt3 pull-down, an increase in the sumoylated Yen1 fraction (≈5-fold) was detected in the *slx8*∆ and *slx5*∆ strains (Fig. 3d). Treatment with the proteasome inhibitor MG132 reduced the differences in the recovery of the sumoylated fractions between the wild type and the *slx5*/*slx8* mutants suggesting that this increase might be due to the combined effects of more DNA damage in the absence of Slx5/Slx8^42^ and decreased removal in these strains of sumoylated proteins^43^, including Yen1.

### *slx8*∆ cells present persistent Yen1 foci

**Fig. 4:**
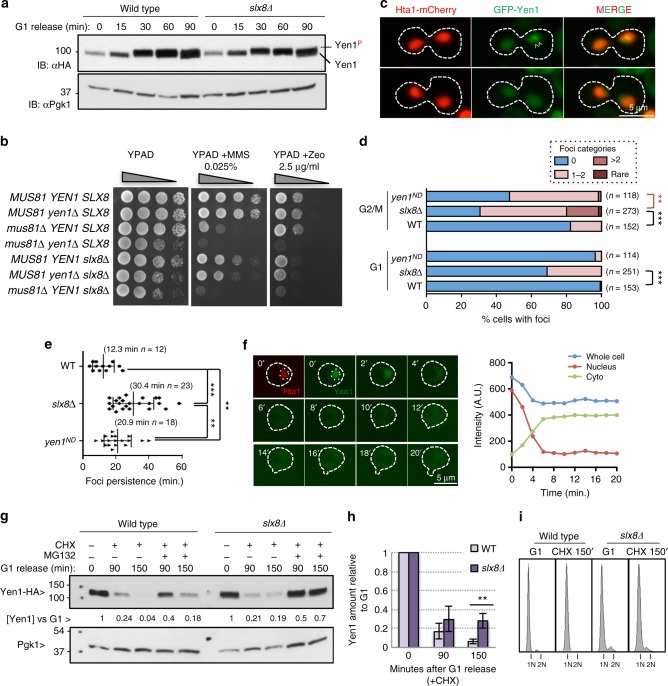
Deletion of *SLX8* alters the nuclear distribution and turnover of a fraction of Yen1. **a** Wild type and *slx8*∆ cells were synchronized in G1 and released to observe the phosphorylation of Yen1 as a function of cell-cycle progression. **b** Serial dilutions of the indicated strains were spotted onto YPAD media containing different genotoxins. **c** Cells with an endogenous *HTA1*-mCherry carrying plasmids expressing wild-type GFP-Yen1 were observed microscopically after a short induction of the fusion protein. Shown are cells presenting normal nuclear localization (lower) or presenting foci (white arrows, upper). **d** G1 and G2/M cells of the indicated genetic backgrounds were microscopically examined as in **c** and classified according to the number of foci they displayed. The graphs show the percentage of cells in each category. The total number of cells individually scored from three video recordings are indicated as (*n*). Categories were subjected to the Fischer’s exact test, asterisks denote significant levels at *P* < 0.001(***) or *P* < 0.005(**). **e** The duration of foci in the indicated genetic backgrounds was measured by video-microscopy analysis. The mean ± s.d. of the duration time and *n* are indicated, error bars denote s.d. Asterisks refer to significance at the *P* < 0.05 (**) and *P* < 0.001 (***) levels in unpaired two-tailed Student’s *t-*test. **f** Cells expressing GFP-Yen1 and Hta1-mCherry were observed by video-microscopy in 2′ time-lapse frames. GFP total intensity of the whole cell, the nucleus and the cytoplasm was determined for 5 z-planes and used to calculate the total GFP intensity in each compartment. The graph displays the time course of GFP intensity in a single cell **g** The indicated *pdr5*∆ strains, that are permeable to MG132, were synchronized in G1 and subjected to cycloheximide (CHX) treatment during their release from G1 arrest. Where indicated, cells were pre-treated with MG132 for 30 min before, and during release in the presence of CHX. PGK1 was used to normalize the amount of Yen1. **h** Quantitation of the fraction of Yen1, compared to G1, remaining at the indicated times after release into CHX. The mean ± s.d. of triplicate assays is shown; statistically significant difference in unpaired two-tailed Student’s *t-*test is indicated (**P < 0.05)**. i** FACS analysis of cells at the beginning and at the end of the CHX treatment

We next addressed the possible impact of Slx5–Slx8 impairment on the dynamics and function of Yen1. The cell-cycle regulation or degree of Yen1 phosphorylation was not grossly altered by deletion of *SLX8* (Fig. 4a). An *slx8*∆ single deletion showed no increase in MMS sensitivity when combined with *yen1*∆, although *yen1*∆ displayed the known synergistic defect when combined to *mus81*∆ (Fig. 4b). A subtle increase in sensitivity for the *slx8*∆ *yen1*∆ mutant was detected with the radiomimetic drug Zeocin, but this was not as pronounced as the synergistic effects of *slx8∆ mus81∆*.

To identify potential defects in the nuclear transport and distribution of Yen1 in the absence of Slx5–Slx8 we GFP-tagged Yen1 at its N-terminus, where we previously obtained positive BiFC data (Fig. 2). After mildly inducing GFP-Yen1 from a plasmid in a strain carrying Hta1-mCherry and a fully functional *YEN1-HA* endogenous allele cells were judged to be healthy and we observed the expected cellular distribution of wild-type Yen1 with its nuclear exclusion occurring at S-phase (Supplementary Figure 2).

Compared to wild type, *slx8*∆ cells were three times more likely to display Yen1 foci (Fig. 4c, Supplementary Figure 2). To characterize the phenotype, cells were classified as containing no focus, 1–2 foci per cell, more than 2 foci and rare events with abnormal features (Fig. 4d, Supplementary Figure 2). The *slx8*∆ cells showed foci of all classes in G2/M, and there was a dramatic increase in cells with 1–2 foci in G1 phase.

Yen1 foci may reflect accumulation of non-degraded or chromatin-associated protein, resulting from faulty nuclease activity or impaired turnover. We compared foci formation in wild type and *slx8*∆ cells to those formed in cells carrying the nuclease dead Yen1^E193A,E195A^ (Yen1^ND^). As expected, Yen1^ND^ also showed higher foci accumulation compared to wild type suggesting that GFP-Yen1 reflects, at least partially, dynamics of an active protein. However, Yen1^ND^ did not accumulate foci in G1 as observed in *slx8*∆ cells (Fig. 4d). We used video microscopy to determine the duration of the foci before they were dispersed. The *slx8*∆ Yen1 foci were about three times more stable (30.4 min) than those detected in wild-type cells (12.3 min) and 50% more stable than Yen1^ND^ (20.9 min) (Fig. 4e). This supports the idea that foci accumulation due to faulty nuclease activity is different from that detected in a strain devoid of Slx5/8. The average intensity of these foci, compared to the nuclear average intensity, was not increased between the different strains.

A closer analysis by video microscopy time-lapses allowed us to observe the disappearance of nuclear signal in cells morphologically in G1 by following the intensity of GFP in both the nuclear and cytoplasmic compartments (Fig. 4f, Supplementary Figure 3). Although most of the signal present in the nucleus was transferred and diluted into the cytoplasm we observed a decline in overall signal during cytoplasmic re-localization (Fig. 4f, Supplementary Figure 3). This suggests that a wave of degradation occurs in a small window of time after nuclear exclusion (at the G1 to S transition). Compared to cells with no focus at the G1-S transition, cells with a focus showed increased nuclear signal for a larger time suggesting its rate of re-localization and degradation is altered (Supplementary Figure 3).

The Slx5–Slx8 Ub ligase is known to target substrates that are subject to stage-specific degradation as well as constitutive turnover^27^. Thus, a possible explanation for the accumulation of Yen1 in foci that persist until G1 in the *slx8*∆ mutant, is that Slx5–Slx8 targets only a fraction of Yen1. To test this hypothesis we further determined whether Yen1 is degraded at the G1-S transition as suggested by the microscopy time-lapse experiments by performing a cycloheximide chase experiment on cells synchronized in G1 (Fig. 4g, h, i). Under these conditions, we detected the disappearance of Yen1 following release from G1 (Fig. 4g). The degradation of Yen1 was at least partially dependent on the presence of a functional proteasome as MG132 largely prevented the degradation. In the absence of Slx8, 20–30% of Yen1 (relative to its G1 level) persisted 150 min after addition of cycloheximide, a time at which the protein was completely degraded in wild-type cells. These data show that whereas Slx5–Slx8 plays a role in Yen1 turnover, it is not the only pathway targeting Yen1 for degradation.

### Yen1 localizes to the nucleolus in the absence of DNA damage

It has been previously reported that *slx8*∆ induces a larger amount of DNA damage by interfering with the control of multiple targets associated with DNA repair^42^. Slx5–Slx8 co-localizes to sites of stalled replication or to Rad52 foci^42^, suggesting that its deletion will impair the normal function of replication and recombination and generate multiple sites of damage. Contrary to this view, we detected only 1–2 Yen1 foci in the majority of *slx8*∆ cells; this result suggests that Yen1 clusters specifically in a nuclear region that may experience more spontaneous damage.

**Fig. 5:**
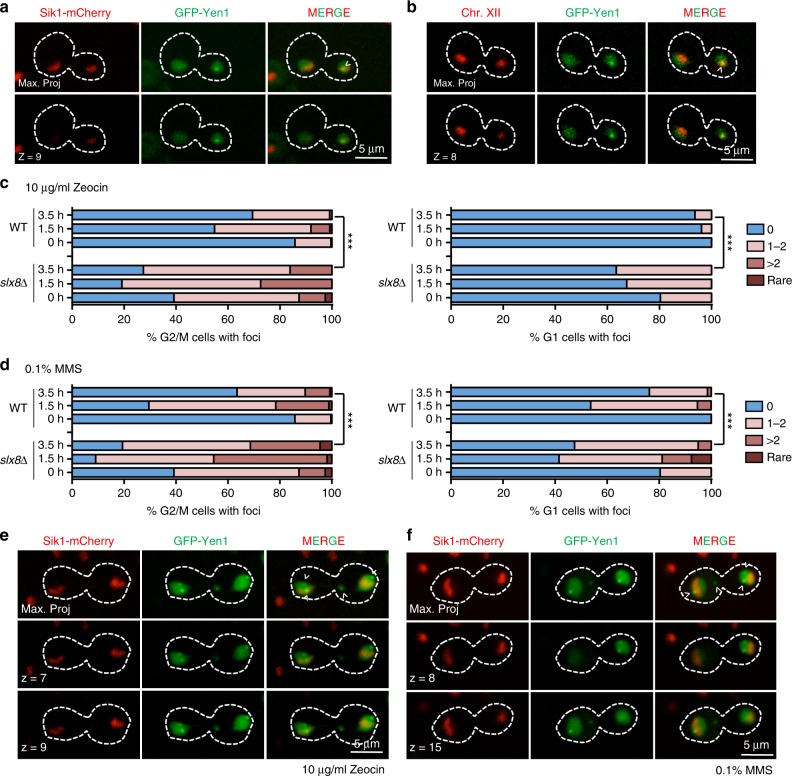
Yen1 foci are dynamic and localize preferentially to nucleolar sites in the absence of DNA damage. **a** *slx8***∆** cells carrying a *SIK1*-mCherry endogenous marker and an inducible GFP-Yen1 expressing plasmid were observed after short induction of the fusion protein. The white arrow denotes co-localizing signal of GFP-Yen1 with Sik1-mCherry. **b** Wild-type cells carrying a TetO-TetR array tag on chromosome XII and an inducible GFP-Yen1 expressing plasmid were observed after short induction of the fusion protein. **c** Cells were subjected to acute challenge with Zeocin (0.01 mg/ml) and observed during their recovery as in Fig. 4. Cells displaying the designated categories of GFP-Yen1 foci were scored at the indicated time points. The total number of cells analysed (*n*) from two independent recordings were as follows: WT 0 h (*n*_G1_ = 81, *n*_G2/M_ = 267), WT 1.5 h (*n*_G1_ = 78, *n*_G2/M_ = 124), WT 3.5 h (*n*_G1_ = 110, *n*_G2/M_ = 118), *slx8*∆ 0 h (*n*_G1_ = 107, *n*_G2/M_ = 79), *slx8*∆ 1.5 h (*n*_G1_ = 40, *n*_G2/M_ = 73), *slx8*∆ 3.5 h (*n*_G1_ = 52, *n*_G2/M_ = 62). **d** Cells were subjected to an acute challenge with 0.1% MMS and for foci were observed as in **c**. The total number of cells analysed (*n*) from two independent recordings were as follows: WT 0 h (*n*_G1_ = 81, *n*_G2/M_ = 267), WT 1.5 h (*n*_G1_ = 95, *n*_G2/M_ = 98), WT 3.5 h (*n*_G1_ = 139, *n*_G2/M_ = 137), *slx8*∆ 0 h (*n*_G1_ = 107, *n*_G2/M_ = 79), *slx8*∆ 1.5 h (*n*_G1_ = 53, *n*_G2/M_ = 55), *slx8*∆ 3.5 h (*n*_G1_ = 40, *n*_G2/M_ = 67). **e** *slx8*∆ cells carrying *SIK1*-mCherry were observed after Zeocin challenge to determine GFP-Yen1 co-localization. White arrows indicate GFP-Yen1 foci. **f** *slx8*∆ cells were observed as in **e**, but were subjected to MMS treatment. White arrows indicate GFP-Yen1 foci. Images are representative of the reproducible results obtained after three independent trials. Statistical significance at *P* < 0.0001 in Fischer’s exact test at 3.5 h recovery points is indicated by asterisks in **c** and **d**

We speculated that Yen1 accumulates in the absence of exogenous DNA damage in the nucleolus, where rDNA loci often generate DNA structures that are substrates for Yen1 activity^8^. Indeed, most of the Yen1 foci detected in *slx8*∆ cells, where foci appeared in a large fraction of cells, co-localized with the Sik1 and Nop1 nucleolar markers (Fig. 5a, Supplementary Figure 4). In wild-type cells, the foci also accumulated in the nucleolus and also localized adjacent to the rDNA array on chromosome XII (Fig. 5b), indicating that Yen1 normally resides there. Slx5–Slx8 localizes to nucleolar sites^42^, and accordingly, we often detected interaction by BiFC of Slx5 and Yen1 adjacent to nucleolar stained regions (Supplementary Figure 4b). Interestingly, we also detected Yen1 foci associated with lagging chromatin between the nuclear masses in a number of *slx8*∆ cells (Supplementary Figure 4c). This suggests that Yen1 is associated with chromatin regions that are having difficulty segregating, where the nuclease may act to resolve joint-molecule intermediates.

After Zeocin or MMS treatment, wild type and *slx8*∆ cells accumulated foci in larger numbers than untreated cells. But in *slx8*∆ cells, many of these foci failed to diffuse significantly after 3.5 h (Fig. 5c, d). Furthermore, when *slx8*∆ cells carrying a Sik1 nucleolar marker were treated with Zeocin or MMS we found that some foci induced after the drug challenge de-localized from the nucleolus (Fig. 5e, f, Supplementary Figure 4), demonstrating that foci are dynamic and can be formed at other undetermined nuclear sites.

### Lysine^714^ is the main Slx5/8 target in Yen1

In order to identify the lysine residues in Yen1 that are targeted by Slx5–Slx8, we performed a mass-spectrometry of immunoprecipitated Yen1-3xFLAG following exposure of cells to MMS. We recovered a peptide harboring a modification consistent with the presence of ubiquitin on the lysine 714. Although the score was below the level of significance, we generated an endogenous replacement with the allele *yen1-K714R-*HA.

**Fig. 6:**
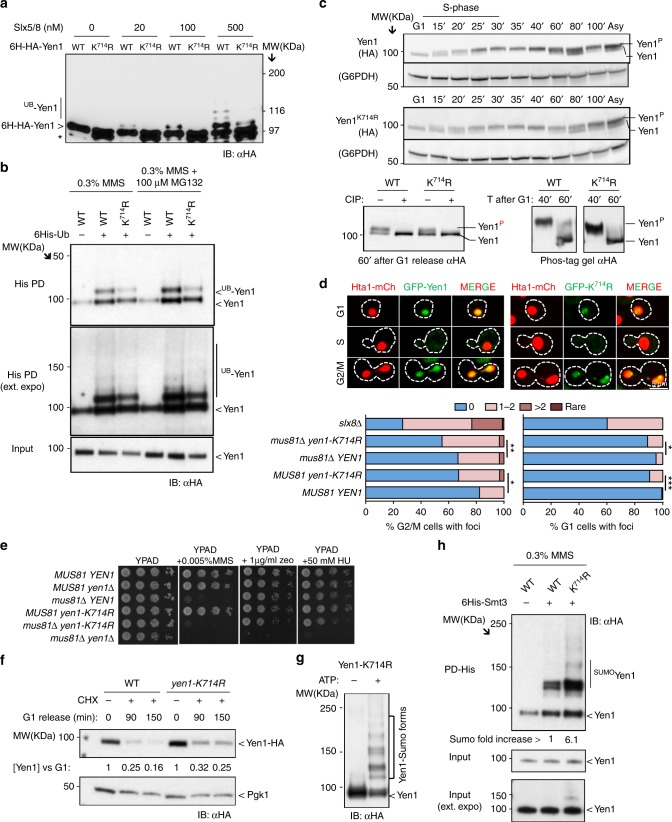
K714 is ubiquitinated by Slx5–Slx8. **a** Recombinant 6xHIS-HA-Yen1 and the mutant 6xHIS-HA-Yen1-K714R (916 nM) were subjected to in vitro ubiquitination as in Fig. 3a. **b** 6xHis-Ubiquitin pull-downs were performed on cells expressing 6His-Ub and carrying endogenous Yen1-HA or its variant Yen1-K714R-HA following treatment with the indicated genotoxics. **c** Strains carrying endogenous Yen1-HA or its K714R variant were synchronized with alpha factor in G1 and proteins extracted at indicated time points after G1 release and immunoblotted with anti-HA. At time points where Yen1 is modified by CDK1, extracts were subjected to phosphatase treatment (CIP+) and also subjected to phos-tag gel separation. **d** Strains carrying *HTA1*-mCherry and the indicated GFP-Yen1 expression plasmids were examined microscopically as in Fig. 4c to assess the presence of the proteins. Foci were quantified as a function of cell-cycle phase, which was determined by cell morphology. The total number of analysed cells (*n*) and independent video recordings (VR) were as follows: WT (*n*_G1_ = 105, *n*_G2/M_ = 153, VR = 3), *yen1*-K714R (*n*_G1_ = 106, *n*_G2/M_ = 64, VR = 3), *mus81*∆ (*n*_G1_ = 294, *n*_G2/M_ = 337, VR = 3), *mus81*∆ *yen1*-K714R (*n*_G1_ = 203, *n*_G2/M_ = 306, VR = 3), *slx8*∆ (*n*_G1_ = 166, *n*_G2/M_ = 172, VR = 3). Statistical differences were estimated by the Fischer’s exact test and significance is indicated by asterisks *P* < 0.05(*), *P* < 0.005(**), *P* < 0.001(***). **e** Sensitivity to the indicated genotoxics was determined by spotting serial dilutions of different strains on the indicated media. **f** Cycloheximide chase experiment showing persistence of Yen1 after a G1 release in the presence of CHX. **g** Immunoprecipitated Yen1-K714R-HA was eluted and the protein was sumoylated with Aos1-Uba2, Ubc9, and Smt3-3KR in the presence or absence of ATP. Samples were de-phosphorylated with CIP before loading. **h** 6xHIS-Smt3 pull-downs were performed on cells expressing 6HIS-Smt3 and carrying *YEN1-HA* or its variant *yen1-K714R-HA* under conditions of MMS treatment as indicated. The average fold enrichment is indicated at the bottom of the blot

Our previous experiments with ubiquitin pull-downs showed that Yen1 was not exclusively ubiquitinated by Slx5–Slx8 in vivo (Fig. 3c). Therefore, we tested the lysine mutant by in vitro ubiquitination using Slx5–Slx8 and recombinant Yen1-K714R. As shown in Fig. 6a, the K714R mutation prevented Yen1 ubiquitination in vitro, supporting the identification of K714 as a target for the ubiquitin ligase. As expected, pull-down analyses with the strains harboring Yen1^K714R^ did not eliminate the recovery of ubiquitinated Yen1. This is likely due to the presence of overlapping pathways of ubiquitin-mediated turnover of Yen1. However we can nonetheless see a decrease in overall ubiquitination in the K714R mutant (Fig. 6b).

### Foci distribution and sumoylation in *yen1-K714R cells*

To determine the effect of the K714R mutation, we initially assessed its cell-cycle regulated localization and activity. Yen1 is phosphorylated by Cdc28 (Cdk1) in S-phase and is de-phosphorylated by Cdc14 at anaphase^44^. No major difference could be detected when analyzing the mutant *yen1-K714R* as compared to wild type (Fig. 6c, Supplementary Figure 5). Nuclear shuttling of Yen1^K714R^ was identical to wild-type Yen1, with S-phase exclusion occurring in both strains (Fig. 6d). Interestingly, we detected a modest increase in Yen1^K714R^ foci in undamaged conditions, further increased in a *mus81*∆ background (Fig. 6d), suggesting the persistence of Yen1^K714R^ foci is specifically observed if there are increased substrates. This increase in foci in the *yen1-K714R mus81*∆ double mutant did not result in increased DNA damage sensitivity (Fig. 6e, Supplementary Figure 5), however, suggesting that locally accumulated Yen1^K714R^ retained the ability to complement the loss-of-Mus81 nuclease. A cycloheximide chase experiment with the *yen1-K714R* strain resulted in the detection of a more persistent Yen1 fraction 150 min after G1 release (Fig. 6f) that is in agreement with the observation of increased Yen1 foci. Yen1-K714R was still sumoylated both in in vitro assays with immunoprecipitated Yen1-HA or in pull-down experiments (Fig. 6g, h). The modified forms recovered from pull-downs were more abundant in the mutant strain compared to the wild type suggesting that the lysine substitution triggers stabilization of the Yen1 sumoylated fraction similarly to the effects observed in the experiments with *slx8*∆ and *slx5*∆ cells (Fig. 3).

### Increased crossover formation in *yen1-K714R* cells

**Fig. 7:**
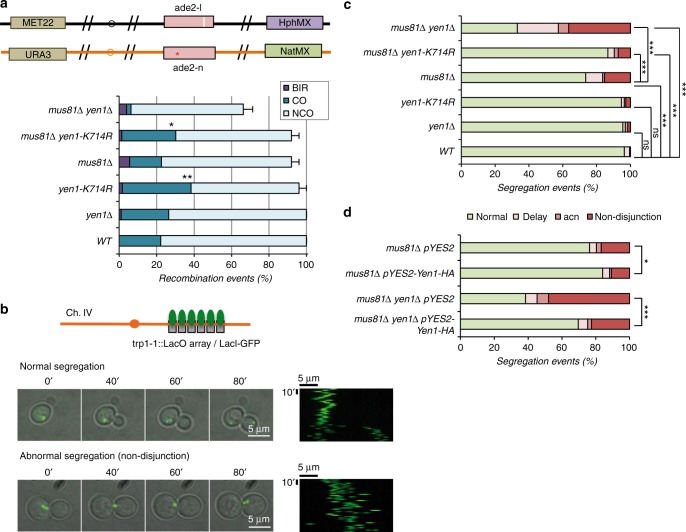
Yen1-K714R increases COs and suppresses spontaneous chromosome segregation defects in *mus81*∆ cells. **a** Diagram explaining the Chr. XV DSB-induced crossover reporters. Recombination outcomes were scored in white/red sectored colonies of the indicated strains and normalized to its plating efficiency (PE). The number of independent experiment trials (T) and the total number of recombination events scored (*n*) were as follows: WT (*T* = 6, *n* = 158), *yen1*∆ (*T* = 9, *n* = 538), *mus81*∆ (*T* = 5, *n* = 168), *mus81*∆ *yen1*∆ (*T* = 9, *n* = 160), *yen1*-K714R (*T* = 5, *n* = 230), and *mus81*∆ *yen1*-K714R (*T* = 5, *n* = 230). **b** A strain harboring a lacO/GFP-LacI array tag on chromosome IV was followed by video-microscopy to discriminate chromosome segregation in timely manner from aberrant segregation. Images display a typical normal and aberrant segregation and its respective kymograph. **c** GFP foci of the indicated strains were observed by video-microscopy and chromosomal segregation was scored as to whether it displayed a proper phenotype (normal) or one of three types of defective phenotypes (non-disjunction, delay, and aberrant chromosome number (acn)). The total number of cells analysed (*n*) and independent video-recordings (VR) were as follows: WT (*n* = 606, VR = 3), *yen1*∆ (*n* = 133, VR = 3), *yen1*-K714R (*n* = 131, VR = 3), *mus81*∆ (*n* = 259, VR = 5), *yen1*-*K714R mus81*∆ (*n* = 448, VR = 3) and *yen1∆ mus81∆ (n* = 289, VR = 3). **d** Segregation events scored as in **c** were determined for *mus81*∆ and *mus81∆ yen1∆* strains containing a pYES2 plasmid expressing Yen1-HA under Galactose-inducible control or an empty pYES2 and subjected to acute over-expression of Yen1-HA or the equivalent mock induction prior to the recording of the video-microscopy. The total number of cells analysed from three VRs were as follows: *mus81*∆ (+pYES2) *n* = 245, *mus81*∆ (+pYES2-Yen1) *n* = 391, *mus81∆ yen1∆* (+pYES2) *n* = 117, *mus81*∆ *yen1*∆ (+pYES2-Yen1) *n* = 218. Statistically significant differences in **a** between CO and other outcomes and in **c** and **d** between normal and abnormal categories were determined by the Fischer’s exact test, asterisks refer to significance at the *P* < 0.001(***), *P* < 0.005(**) or *P* < 0.05(*)

To test possible gain-of-function effects of Yen1^K714R^ we measured mitotic CO levels. A gain-of-function of Yen1 would likely increase the rate of CO resolution during DSB repair without affecting sensitivity to DNA damaging agents. To test this hypothesis, we used the diploid strain able to screen CO/BIR levels after an I-SceI-induced DSB^45^. Introduction of -1xHA tagged Yen1 in these strains did not affect the CO levels previously reported^46^ and were within experimental variation. As expected for this assay, the *mus81*∆ strain showed a slightly reduced CO level that was further reduced in the *mus81∆ yen1∆* strain, along with a parallel increase in BIR (Fig. 7a). Surprisingly, when introduced alone, Yen1^K714R^ increased the overall CO levels above those of wild type. Further, when *yen1-K714R* was combined with *mus81*∆, instead of a decrease in CO levels as expected for a loss-of-function *YEN1* allele, we observed a nearly twofold increase in COs compared to *mus81*∆ alone (Fig. 7a). These CO levels are above those expected for wild type and consistent with a gain-of-function phenotype.

### *yen1-K714R* suppresses segregation defects of *mus81∆*

One effect of the failure to repair recombination intermediates is the accumulation of joint-molecules in mitosis that lead to mis-segregation of chromosomes and ultimately an increase or decrease in chromosome number. The *mus81∆ yen1∆* strain display such segregation defects even in the absence of DSBs induced by exogenous sources^45^. In order to test the *yen1-K714R* mutant for a gain-of-function phenotype, we devised an improved assay for chromosome segregation fidelity. Genetic systems that monitor mis-segregation have the limitation of looking at viable endpoints and cannot recover unviable ones. We therefore chose to look at real-time chromosome segregation by using a fluorescent array tag on chromosome IV (Fig. 7b, Supplementary Figure 6). Using this approach we confirmed that *mus81∆ yen1∆* cells are severely impaired in chromosome segregation (Fig. 7c). Segregation defects were observed in 35% of *mus81*∆ single mutants, and more than 60% of *mus81*∆ *yen1*∆ cells (Fig. 7c). Under the same conditions, *yen1*-K714R partially suppressed the *mus81*∆ defects (Fig. 7c). The ability of Yen1^K714R^ to suppress *mus81*∆ defects is further evidence of a gain-of-function phenotype consistent with the idea that more spontaneous joint-molecule intermediates are resolved in cells expressing Yen1^K714R^ than in wild-type cells. Because elevated Yen1 expression has been shown to suppress *mus81Δ* phenotypes^47^, we compared the effect of Yen1 over-expression to the phenotypes of the *yen1-K714R* mutant. Expression of plasmid borne wild-type Yen1-HA, suppressed *mus81Δ* segregation defects to levels similar to those observed in a *mus81Δ yen1-K714R* strain (Fig. 7d). Thus, even though Yen1-HA protein levels were extremely high following induction (see Supplementary Figure 7b), suppression was no better than that achieved by endogenous levels of Yen1^K714R^. Interestingly, long-term high-level expression of Yen1 appeared to be deleterious to *mus81Δ* cells. Chronic but mild over-expression of Yen1-HA (0.1% galactose induction) resulted in *mus81*∆ cells that were more sensitive to MMS than their un-induced counterparts even though Yen1-HA expression was only slightly higher than endogenous levels (Supplementary Figure 7).

## Discussion

Nucleolytic processing of recombination intermediates by Yen1 has been proposed to be an option of last resort that is needed to prevent chromosomal segregation defects and genome rearrangements in mitosis^48^. Accordingly, Yen1 has a tightly controlled operational window that is limited to the anaphase—telophase period of mitosis. Here, it removes the last intermediates that physically connect the chromosomes^44,49^. Although the window of Yen1 activity is known to rely on cell-cycle driven phosphorylation and de-phosphorylation^12,13^, we establish here for the first time a new layer of control involving Slx5–Slx8 dependent ubiquitination of Yen1 (see model in Supplementary Figure 8). Slx5/8 targets a minor fraction of Yen1 that is detected as a more persistent pool associated with nucleolar or active sites on the chromatin (Figs. 4 and 5). We have identified K714 as the major target of Slx5–Slx8 (Fig. 6). The fact that significant levels of ubiquitinated Yen1 were recovered from *slx8*∆ cells, is in full agreement with reports that other Slx5/8 targets are redundantly targeted by alternative ubiquitination pathways to ensure rapid destruction in different contexts or sub-nuclear compartments^41,50,51^.

Our analysis of the transition between G1 and S phases clearly indicates that there is a wave of Yen1 destruction before S-phase entry, when most of the previously nuclear protein is targeted for proteasomal degradation (Fig. 4). Our data suggest this is a general turnover pathway that is not controlled by Slx5/8. This pathway degrades most of the Yen1 pool, similar to what has been described for other Slx5/8 targets^51^. One plausible explanation for why Slx5/8 targets such a minor fraction of Yen1 is that it can be quickly degraded, or evicted from the nucleus before S-phase starts. In agreement with such a hypothesis, we see a fraction of Yen1 that remains un-degraded in cycloheximide chase experiments performed in cells that are synchronized in G1. The amount of Yen1 that remains un-degraded is about 20% of the total found at the beginning of the experiment, although this may reflect an increased amount of Yen1 associated with chromatin or active sites in cells devoid of Slx5/8^42,52^. Under wild-type conditions, we might expect the fraction of Yen1 being targeted by Slx5/8 to be more transient. Our data are also compatible with a SUMO-independent role of Slx5/8 in targeting Yen1 (Fig. 3). SUMO independence has previously been observed in certain Slx5/8 substrates. These substrates are thought to be targeted by Slx5/8 binding to sumoylated partner proteins^40^ or to substrate structural features that mimic SUMO^51^.

Sumoylation has been proposed as a mechanism to rapidly form protein complexes under stress conditions^20^, or to recruit factors that need to associate at precise cellular localizations in a timely constrained manner^53^. We speculate that Yen1 is sumoylated in stressful situations, and that Slx5/8 ubiquitination ensures any remaining Yen1 that is strongly associated with DNA at the end of G1 will be effectively degraded before replication starts. In such a hypothesis, sumoylation would not be a pre-requisite for Slx5/8 targeting, but sumoylated Yen1 would be targeted due to its preferential association with DNA or chromatin regions that preclude it from being exported or degraded at S-phase similar to what has been described for proteins associated with the kinetochore^40^. Although, we consider it unlikely, our data does not exclude the possibility that Slx5/8 ubiquitination does not directly trigger Yen1 degradation, but only influences its local association with other partners or its DNA substrates. For example, the extended nuclear persistence of a limited Yen1 fraction could also result from its association with nuclear sites that are inhibitory to a general mechanism of degradation that has yet to be described and is responsible for eliminating the majority of the Yen1 protein before S-phase.

The fact that Yen1 is regulated by multiple mechanisms, including phosphorylation, nuclear exclusion, and protein turnover, suggests that Yen1 inhibition is especially important for preventing DNA damage during DNA replication when structures generated at the replication forks may be a substrate for Yen1 cleavage. In agreement with this hypothesis, hyperactive forms of Yen1 that are constitutively nuclear (Yen1^ON^) sensitize cells to high doses of MMS^12^. This has been interpreted as an increased sensitivity to conditions where forks stall more frequently. Similarly, we can detect a suppressive effect linked to over-expression of Yen1, but chronic mis-regulation of Yen1 levels sensitizes cells and causes deleterious side effects (Fig. 7, Supplementary Figure 7), whereas we did not detect an increased sensitivity to MMS in *yen1*-K714R cells. It is important to keep in mind that redundant controls are probably able to restrict Yen1 activity if overall levels are not largely altered, but these controls may not be sufficient to cope with a chronic over-expression of the nuclease. With moderate excess of Yen1, the protein may still become dissociated from chromatin and exported prior to S-phase and it can still be phosphorylated by CDK1 to inhibit its activity thus explaining limited impacts in MMS sensitivity. Nonetheless, we see a striking increase in CO formation with the *yen1*-K714R allele following a DSB, suggesting that the Slx5/8 control plays an important role in maintaining the preference for the use of NCO pathways (Sgs1- or Mph1-dependent pathways) whenever these factors are available. It is known that Slx5/8 is needed to allow re-localization of DSBs to nuclear pores^54^ and could thus regulate the ability of repair factors to gain access to the intermediates of DSB repair^54,55^. We suggest that Slx5/8 may be responsible for clearing Yen1 from DNA intermediates where Sgs1- is already recruited, or for limiting Yen1’s ability to associate to these intermediates. The fact that the K714R mutation allows increased recovery of sumoylated Yen1 (Fig. 6) raises the interesting possibility that sumoylation favors Yen1 activity over other proteins capable of dismantling joint-molecule intermediates. Slx5–Slx8 may regulate access of different proteins by removing sumoylated Yen1 from DNA intermediates if its recruited in the presence of NCO determinants like Sgs1. Mapping of the multiple sites of Yen1 sumoylation will be required to determine whether sumoylation is strictly required for Yen1 functions in vivo, and whether different sites or levels of sumoylation influence its binding to known partners, or proteins yet to be described. Should any potential “sumo-less” mutants, bearing multiple lysine replacements, be neutral with respect to Yen1’s protein folding, it would be possible to draw conclusions on the effect of sumoylation on Yen1’s localization and nuclease activitiy. Similarly, a more extended study on the biochemical activities of in vitro fully-sumoylated Yen1 will help test whether sumoylation alters Yen1’s specificity for its different substrates.

Yen1 has a specific role in dealing with replication intermediates that are usually handled by Dna2^38^ and it plays a role in suppressing the accumulation of JM intermediates that originate in the rDNA locus^8^. These JM contain either orphan HJs or D-loop -derived intermediates that do not mature into dHJs, and thus are not dissolved by the BLM ortholog Sgs1 and its associated proteins^4,8^. Slx5/8 could thus ensure that Yen1 is only used as the last option even in situations when recruitment has been enforced by a sumoylation cascade^20^. If the human GEN1 ortholog is regulated similarly, we expect that mutations equivalent to the K714R identified here may recapitulate the hyper-crossover phenotypes we observed without a major effect on cell viability. Such mutations might be found to have a cancer predisposition phenotype due to its increased genome instability. Defects in GEN1 turnover would be expected to give rise to a phenotype similar to that of impaired BLM helicase, or a defect in factors enforcing NCO pathways. The redundancy in the pathways of nucleolytic resolution during HR in humans^56^ makes it difficult to determine a precise role of GEN1 mutations in cancer predisposition, with scarce evidence to date^57–59^. However, should the active pools of GEN1 be similarly regulated in human cells, it may be possible to identify mutations that destabilize the balance between CO and NCO outcomes.

## Methods

### Yeast strains and growth conditions

*S. cerevisiae* strains and plasmids used in this study are listed in Supplementary Tables 1 and 2. The *YEN1*-HA allele was generated by inserting a FactorXa cleavage site and a single-HA epitope at its C-terminus using PCR amplification with a dedicated oligonucleotide. Mutants in the different designated loci where either obtained by crossing or by gene replacement with the indicated selective cassettes. Cells were typically grown in YP (1% yeast extract; 2% peptone) or SC media with 2% glucose or alternatively with 2% raffinose or 2% galactose in strains under inducible conditions. A modified medium (SC with 0.17% YNB without ammonium sulfate, 0.1% proline and 0.003% SDS) was used for the cycloheximide and MG132 assays and for the Smt3/Ubi pull-down assays. Methyl methane sulfonate (MMS, Sigma), Zeocin (Zeo, Invitrogen) and hydroxyurea (HU, Sigma) were added to YPD medium at the designated doses for DNA damage sensitivity assays.

### Western blot analyses

Proteins were extracted by the TCA (Trichloroacetic acid) method if not stated otherwise. Samples were loaded into 7.5% or 4–15% gradient Tris-Glycine BioRad stain-free pre-casted gels for routine analysis. Samples from pull-downs analyses were loaded into 3–8% gradient NuPAGE Tris-Acetate gels (Invitrogen). Gels were transferred to PVDF membranes using a semi-dry transfer machine (BioRad) and hybridized on TBST 5% milk with the appropriate antibodies. Antibodies were used at the suggested dilution: anti-HA-HRP (3F10, Roche) 1:1000 (1:500 for pull-down analysis), anti-Ubiquitin (P4D1, Biolegends) 1:1000, anti-Smt3 (provided by B.Palancade) 1:2000, anti-G6PDH (A9521, Sigma) 1:20,000, anti-Pgk1-HRP (22C5D8, Abcam) 1:10,000, anti-GST-HRP (GERPN1236, Sigma) 1:5000. When required, HRP-conjugated secondary antibodies from Cell Signaling (anti-Mouse-HRP, anti-Rabbit-HRP) were used at 1:10,000 dilution. Western blots were revealed with WesternBright ECL solution (Advansta) or WesternBright Sirius HRP subsrate if required (Advansta). Blots were cropped in order to arrange figures without any lane substitution and conserving the area with immunoblotting signal (examples of uncropped images are available in Supplementary Figure 9).

### Microscopy and cell biology

Live cell imaging was performed with a Spinning Disk Confocal Microscope (CSU-W1, Yokogawa), with an electron multiplying charge device camera (ANDOR Zyla sCMOS) and a ×60/1.35 numerical aperture objective at 30 °C. Cells were mounted on agarose pads as described^60^ and imaging recorded 15 z-sections with 0.5 µm spacing. Image acquisition was performed using Fiji (ImageJ)^61^. Cells were grown in synthetic complete medium without uracil (SC-URA 2% raffinose), GFP-Yen1 was induced in a short burst with 30 min with Galactose at 2%, followed by addition of Glucose at 2%. DNA damage acute exposures (MMS 0.1% or 10 μg/ml Zeocin) lasted 15 min at room temperature following arrest of GFP-Yen1 expression. After the acute DNA damage, cells were washed once with fresh SC-URA 2% glucose and held for 30 min at 30 °C in this medium, whereas aliquots were removed at the indicated times. Cells showing an accumulation of spots were measured at maximum projection of the GFP channel. Statistical analysis was performed using Fisher's exact test to determine the level of significance between two categories and *χ*^2^ to compare more than two categories and consistency between trials.

### Cycloheximide chase experiments

Cultures grown in SC complete modified media (0.1% proline 0.017% YNB w/o ammonium sulfate) were diluted to OD 600 = 0.2 and synchronized with alpha factor (3 µM) for 2 h. At G1 release, cells were treated with cycloheximide (250 µg/ml) in fresh media and when indicated cells were pre-treated with MG132 (100 µg/ml) 30 min before G1 release. At every given time point 1 ml of culture was removed and frozen in presence of 25 mM sodium azide. Proteins were extracted by the TCA method and analyzed by 7.5% SDS-PAGE and western blot.

### Pull-downs and immunoprecipitation

For 6xHIS-Smt3 and 6xHIS-Ubi4 pull-downs, strains containing the expression vectors or the control plasmid were grown in SC-LEU modified medium (0.1% proline, 0.17% YNB without ammonium sulfate). Cells were allowed to grow to OD 600 = 0.3 when CuSO_4_ was added at 100 µM final concentration in a volume of 100 ml. After 1 h MMS was added to 0.3% and cells were collected 3 h later. For cultures inhibited for proteasome degradation MG132 was added to 100 µM 2 h before collecting the cells. Cells where lysed under denaturing conditions and SUMO or ubiquitin-conjugated proteins where isolated basically as described^35,62–64^ with the modifications detailed in Supplementary methods section. In GST-pull-downs cells were lysed in IP buffer (40 mM Tris-HCl (pH 7.5), 10% Glycerol 0.1% NP40 150 mM NaCl) and cleared lysates bound to Glutathione Sepharose (GE Healthcare). After washes, proteins were eluted directly in Laemmli buffer. For the detection of sumoylated Yen1-HA forms by immunoprecipitation cells were lysed in TCA buffer by bead-beating at 4 °C. After centrifugation, precipitated proteins were washed once in cold acetone and then, pellets were resuspended in denaturing-IP buffer (0.5 M Tris-base, 6.5% SDS, 100 mM DTT, and 12% glycerol) and heated during 20 min at 65ºC before centrifugation at 16,000g for 10 min. Each 45 μl of soluble protein  were diluted in 1.5 ml of RIPA buffer (50 mM Tris (pH 7.5), 150 mM NaCl, 5 mM EDTA, and 1% Triton X-100) containing protease inhibitors (EDTA free, Roche) and applied to anti-HA conjugated Agarose (Pierce), the bound fraction was eluted in Urea-loading buffer (8 M urea, 200 mM Tris-HCl (pH 6.8), 1 mM EDTA, 5% SDS, 0.1% bromophenol blue and 1.5% DTT).

### Protein purification and in vitro assays

HIS6-tagged recombinant proteins were produced in *E. coli* BL21-RIL cells as described^65^. Proteins were purified on a 1 ml His-Trap column using an AKTA FPLC (GE Healthcare). The peak fractions were identified by SDS-PAGE, pooled, and dialyzed to buffer A (25 mM Tris-HCl (pH 7.5), 1 mM EDTA, 0.01% NP40, 1 mM DTT, 10% glycerol, and 0.1 mM PMSF) containing 300 mM NaCl and stored at −80 °C. Ub, Uba1, and UbcH5 were obtained from Boston Biochem.

In vitro full reconstituted sumoylation and ubiquitination assays with recombinant 6H-HA-Yen1 were performed in the presence of 20 mM HEPES (pH 7.5), 5 mM MgCl_2_, 2 mM ATP, 5 μM ZnSO_4_, and 0.1 mM DTT. Sumoylation reactions were incubated at 30 °C for 40 min and contained 10 nM Aos1/Uba2, 60 nM Ubc9, 0–10 nM Siz2-V5, 1.5 µM His6-Smt3-G98, and 0.7 µM Yen1 in a total volume of 20 μl. Ubiquitination reactions were incubated at 30 °C for 30 min and contained 10 nM Uba1, 60 nM Ubc5, 0–500 nM Slx5–Slx8, and 1.5 μM Ub in a 20 μl reaction. Where present, Holliday junction DNA, comprised of four 49-nt oligonucleotides, was added at 0.2 µM.

In in vitro Sumoylation assays with Yen1-1xHA from yeast, Yen1 was immunoprecipitated from cell lysates in native IP buffer using anti-HA conjugated Agarose (Pierce) and the protein was eluted with HA peptide (Sigma) competition. Eluates were subjected to SUMO conjugation as described^35^.

### DSB-induced *ade2* recombination assay

The diploid recombination assays were performed basically as described^46^. Briefly, diploid strains containing 2 hetero-alleles of *ade2* are cleaved in its *ade2*-I allele by induction of I-SceI and allowed to repair under non-selective conditions (YPD) to give rise to either ADE2 or *ade2*-n repair products in three types of colonies (red, white, and sectored). Outcomes were scored as in ref. ^46^ by assigning to each colony the recombination events that correspond to the repair of the two sister-chromatids. A more detailed overview is available at Supplementary methods section.

## Electronic supplementary material

Supplementary Information

## Data Availability

The data that support the findings of this study are available from the corresponding author upon reasonable request.

## References

[1] Symington LS, Rothstein R, Lisby M (2014). Mechanisms and regulation of mitotic recombination in Saccharomyces cerevisiae. Genetics.

[2] Szostak JW, Orr-Weaver TL, Rothstein RJ, Stahl FW (1983). The double-strand-break repair model for recombination. Cell.

[3] Bzymek M, Thayer NH, Oh SD, Kleckner N, Hunter N (2010). Double Holliday junctions are intermediates of DNA break repair. Nature.

[4] Mazon G, Symington LS (2013). Mph1 and Mus81-Mms4 prevent aberrant processing of mitotic recombination intermediates. Mol. Cell.

[5] Prakash R (2009). Yeast Mph1 helicase dissociates Rad51-made D-loops: implications for crossover control in mitotic recombination. Genes Dev..

[6] Ira G, Malkova A, Liberi G, Foiani M, Haber JE (2003). Srs2 and Sgs1-Top3 suppress crossovers during double-strand break repair in yeast. Cell.

[7] Mitchel K, Zhang H, Welz-Voegele C, Jinks-Robertson S (2010). Molecular structures of crossover and noncrossover intermediates during gap repair in yeast: implications for recombination. Mol. Cell.

[8] Garcia-Luis J, Machin F (2014). Mus81-Mms4 and Yen1 resolve a novel anaphase bridge formed by noncanonical Holliday junctions. Nat. Commun..

[9] Matos J, Blanco MG, Maslen S, Skehel JM, West SC (2011). Regulatory control of the resolution of DNA recombination intermediates during meiosis and mitosis. Cell.

[10] Gallo-Fernandez M, Saugar I, Ortiz-Bazan MA, Vazquez MV, Tercero JA (2012). Cell cycle-dependent regulation of the nuclease activity of Mus81-Eme1/Mms4. Nucleic Acids Res..

[11] Szakal B, Branzei D (2013). Premature Cdk1/Cdc5/Mus81 pathway activation induces aberrant replication and deleterious crossover. EMBO J..

[12] Blanco MG, Matos J, West SC (2014). Dual control of Yen1 nuclease activity and cellular localization by Cdk and Cdc14 prevents genome instability. Mol. Cell.

[13] Eissler CL (2014). The Cdk/cDc14 module controls activation of the Yen1 holliday junction resolvase to promote genome stability. Mol. Cell.

[14] Johnson ES (2004). Protein modification by SUMO. Annu. Rev. Biochem..

[15] Flotho A, Melchior F (2013). Sumoylation: a regulatory protein modification in health and disease. Annu. Rev. Biochem..

[16] Johnson ES, Gupta AA (2001). An E3-like factor that promotes SUMO conjugation to the yeast septins. Cell.

[17] Takahashi Y, Kahyo T, Toh EA, Yasuda H, Kikuchi Y (2001). Yeast Ull1/Siz1 is a novel SUMO1/Smt3 ligase for septin components and functions as an adaptor between conjugating enzyme and substrates. J. Biol. Chem..

[18] Zhao X, Blobel G (2005). A SUMO ligase is part of a nuclear multiprotein complex that affects DNA repair and chromosomal organization. Proc. Natl Acad. Sci. USA.

[19] Nie M, Boddy MN (2016). Cooperativity of the SUMO and ubiquitin pathways in genome stability. Biomolecules.

[20] Psakhye I, Jentsch S (2012). Protein group modification and synergy in the SUMO pathway as exemplified in DNA repair. Cell.

[21] Sarangi P, Zhao X (2015). SUMO-mediated regulation of DNA damage repair and responses. Trends Biochem. Sci..

[22] Sacher M, Pfander B, Hoege C, Jentsch S (2006). Control of Rad52 recombination activity by double-strand break-induced SUMO modification. Nat. Cell Biol..

[23] Pfander B, Moldovan GL, Sacher M, Hoege C, Jentsch S (2005). SUMO-modified PCNA recruits Srs2 to prevent recombination during S phase. Nature.

[24] Hoege C, Pfander B, Moldovan GL, Pyrowolakis G, Jentsch S (2002). RAD6-dependent DNA repair is linked to modification of PCNA by ubiquitin and SUMO. Nature.

[25] Cremona CA (2012). Extensive DNA damage-induced sumoylation contributes to replication and repair and acts in addition to the mec1 checkpoint. Mol. Cell.

[26] Bonner JN (2016). Smc5/6 mediated sumoylation of the Sgs1-Top3-Rmi1 complex promotes removal of recombination intermediates. Cell Rep..

[27] Sriramachandran AM, Dohmen RJ (2014). SUMO-targeted ubiquitin ligases. Biochim. Biophys. Acta.

[28] Prudden J (2007). SUMO-targeted ubiquitin ligases in genome stability. EMBO J..

[29] Ii T, Fung J, Mullen JR, Brill SJ (2007). The yeast Slx5-Slx8 DNA integrity complex displays ubiquitin ligase activity. Cell Cycle.

[30] Xie Y (2007). The yeast Hex3.Slx8 heterodimer is a ubiquitin ligase stimulated by substrate sumoylation. J. Biol. Chem..

[31] Uzunova K (2007). Ubiquitin-dependent proteolytic control of SUMO conjugates. J. Biol. Chem..

[32] Zhang Z, Buchman AR (1997). Identification of a member of a DNA-dependent ATPase family that causes interference with silencing. Mol. Cell Biol..

[33] Wang Z, Jones GM, Prelich G (2006). Genetic analysis connects SLX5 and SLX8 to the SUMO pathway in Saccharomyces cerevisiae. Genetics.

[34] Mullen JR, Kaliraman V, Ibrahim SS, Brill SJ (2001). Requirement for three novel protein complexes in the absence of the Sgs1 DNA helicase in Saccharomyces cerevisiae. Genetics.

[35] Bretes H (2014). Sumoylation of the THO complex regulates the biogenesis of a subset of mRNPs. Nucleic Acids Res..

[36] Parker JL (2008). SUMO modification of PCNA is controlled by DNA. EMBO J..

[37] Altmannova V (2010). Rad52 SUMOylation affects the efficiency of the DNA repair. Nucleic Acids Res..

[38] Olmezer G (2016). Replication intermediates that escape Dna2 activity are processed by Holliday junction resolvase Yen1. Nat. Commun..

[39] Sung MK, Huh WK (2007). Bimolecular fluorescence complementation analysis system for in vivo detection of protein-protein interaction in Saccharomyces cerevisiae. Yeast.

[40] Schweiggert J, Stevermann L, Panigada D, Kammerer D, Liakopoulos D (2016). Regulation of a spindle positioning factor at kinetochores by SUMO-targeted ubiquitin ligases. Dev. Cell.

[41] Hickey CM, Xie Y, Hochstrasser M (2018). DNA binding by the MATalpha2 transcription factor controls its access to alternative ubiquitin-modification pathways. Mol. Biol. Cell.

[42] Burgess RC, Rahman S, Lisby M, Rothstein R, Zhao X (2007). The Slx5-Slx8 complex affects sumoylation of DNA repair proteins and negatively regulates recombination. Mol. Cell. Biol..

[43] Thu YM (2016). Slx5/Slx8 promotes replication stress tolerance by facilitating mitotic progression. Cell Rep..

[44] Matos J, West SC (2014). Holliday junction resolution: regulation in space and time. DNA Repair.

[45] Ho CK, Mazon G, Lam AF, Symington LS (2010). Mus81 and Yen1 promote reciprocal exchange during mitotic recombination to maintain genome integrity in budding yeast. Mol. Cell.

[46] Mazon G, Lam AF, Ho CK, Kupiec M, Symington LS (2012). The Rad1-Rad10 nuclease promotes chromosome translocations between dispersed repeats. Nat. Struct. Mol. Biol..

[47] Munoz-Galvan S (2012). Distinct roles of Mus81, Yen1, Slx1-Slx4, and Rad1 nucleases in the repair of replication-born double-strand breaks by sister chromatid exchange. Mol. Cell Biol..

[48] Matos J, Blanco MG, West SC (2013). Cell-cycle kinases coordinate the resolution of recombination intermediates with chromosome segregation. Cell Rep..

[49] Talhaoui I, Bernal M, Mazon G (2016). The nucleolytic resolution of recombination intermediates in yeast mitotic cells. FEMS Yeast Res..

[50] Rubenstein EM, Hochstrasser M (2010). Redundancy and variation in the ubiquitin-mediated proteolytic targeting of a transcription factor. Cell Cycle.

[51] Xie Y, Rubenstein EM, Matt T, Hochstrasser M (2010). SUMO-independent in vivo activity of a SUMO-targeted ubiquitin ligase toward a short-lived transcription factor. Genes Dev..

[52] Cook CE, Hochstrasser M, Kerscher O (2009). The SUMO-targeted ubiquitin ligase subunit Slx5 resides in nuclear foci and at sites of DNA breaks. Cell Cycle.

[53] Jentsch S, Psakhye I (2013). Control of nuclear activities by substrate-selective and protein-group SUMOylation. Annu. Rev. Genet..

[54] Horigome C (2016). PolySUMOylation by Siz2 and Mms21 triggers relocation of DNA breaks to nuclear pores through the Slx5/Slx8 STUbL. Genes Dev..

[55] Bohm S, Mihalevic MJ, Casal MA, Bernstein KA (2015). Disruption of SUMO-targeted ubiquitin ligases Slx5-Slx8/RNF4 alters RecQ-like helicase Sgs1/BLM localization in yeast and human cells. DNA Repair.

[56] Sarbajna S, Davies D, West SC (2014). Roles of SLX1-SLX4, MUS81-EME1, and GEN1 in avoiding genome instability and mitotic catastrophe. Genes Dev..

[57] Spinella JF (2015). Whole-exome sequencing of a rare case of familial childhood acute lymphoblastic leukemia reveals putative predisposing mutations in Fanconi anemia genes. BMC Cancer.

[58] Kuligina E (2013). Value of bilateral breast cancer for identification of rare recessive at-risk alleles: evidence for the role of homozygous GEN1 c.2515_2519delAAGTT mutation. Fam. Cancer.

[59] Medves S (2016). A high rate of telomeric sister chromatid exchange occurs in chronic lymphocytic leukaemia B-cells. Br. J. Haematol..

[60] Tran PT, Paoletti A, Chang F (2004). Imaging green fluorescent protein fusions in living fission yeast cells. Methods.

[61] Schindelin J (2012). Fiji: an open-source platform for biological-image analysis. Nat. Methods.

[62] Johnson ES, Blobel G (1999). Cell cycle-regulated attachment of the ubiquitin-related protein SUMO to the yeast septins. J. Cell Biol..

[63] Ulrich HD, Davies AA (2009). In vivo detection and characterization of sumoylation targets in Saccharomyces cerevisiae. Methods Mol. Biol..

[64] Rouviere JO (2018). A SUMO-dependent feedback loop senses and controls the biogenesis of nuclear pore subunits. Nat. Commun..

[65] Mullen JR, Brill SJ (2008). Activation of the Slx5-Slx8 ubiquitin ligase by poly-small ubiquitin-like modifier conjugates. J. Biol. Chem..

