# Supplementary material for: Slx5-Slx8 ubiquitin ligase targets active pools of the Yen1 nuclease to limit crossover formation
Source: Nat Commun. 2018 Nov 27;9:5016. doi: 10.1038/s41467-018-07364-x (PMC6258734; doi:10.1038/s41467-018-07364-x)
Supplement: Supplementary file 1 — Supplementary Information [file 41467_2018_7364_MOESM1_ESM.pdf]

Supplementary Information

Slx5-Slx8 Ubiquitin Ligase targets active pools of the Yen1 nuclease to limit crossover formation.

Talhaoui et al.

Supplementary Figures 1-9

Supplementary Tables 1-2

Supplementary Methods

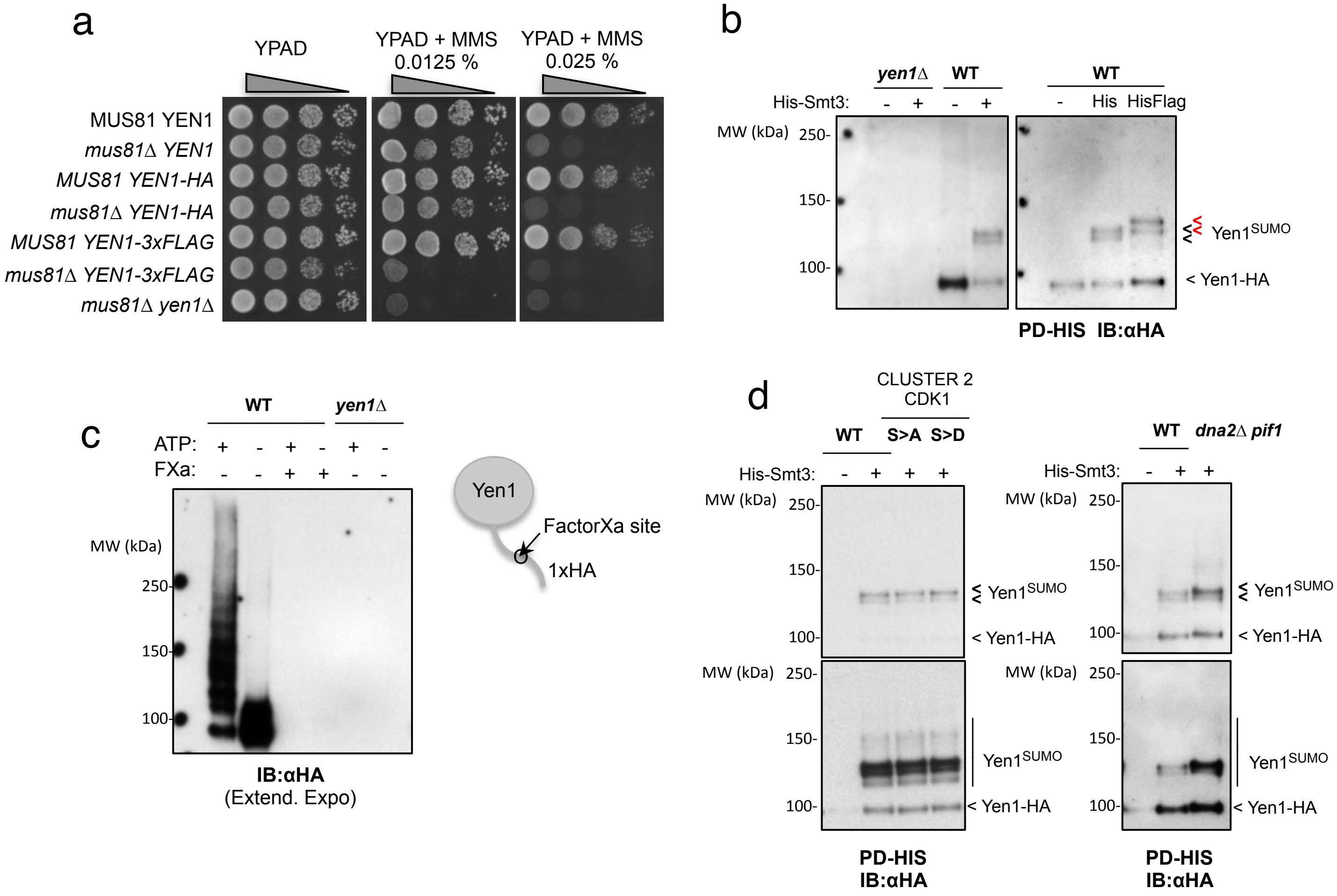

Supplementary Figure 1. **a**) Strains carrying either a wild-type YEN1 locus or an endogenous replacement with the epitope tagged YEN1-HA or YEN1-3xFLAG were combined to *mus81*Δ and sensitivity to MMS was monitored with growth of serial dilutions in plates with the indicated doses of MMS. **b)** Smt3 denaturing pull-down under MMS treatment (0.3%) was performed from either a *yen1*Δ or wild-type strain expressing either empty, 6xHis-Smt3 (His) or His-Flag-Smt3 (HisFlag), small amont of PD was loaded to allow detection of the different bands of Yen1 sumoylation without saturation (dark marks for his-Smt3 or red marks for his-flag-Smt3) **c)** Yen1-HA was overexpressed in wild-type asynchronous cells and equivalent growth was made in an empty *yen1*Δ strain, extracts were immuno-precipitated with anti-HA, eluted by HA peptide competition and mixed with Aos1-Uba2, Ubc9 and Smt3-3KR in the presence or absence of ATP. When indicated, reactions were subjected to Factor Xa (FXa) treatment to remove the –HA tag. **d)** Smt3 denaturing pull-down under MMS treatment (0.3%) was performed from a wild-type strain and strains containing the Alanine or Aspartic substitution of four Serines of the CDK1 sites of cluster 2 (Eissler et al., 2014). Second gel compares wild-type with *dna2*Δ *pif1* strain that results in an average (N=2) fold increase of 3.3 ±0.5 (SD) in the sumoylated fraction for the mutant strain.

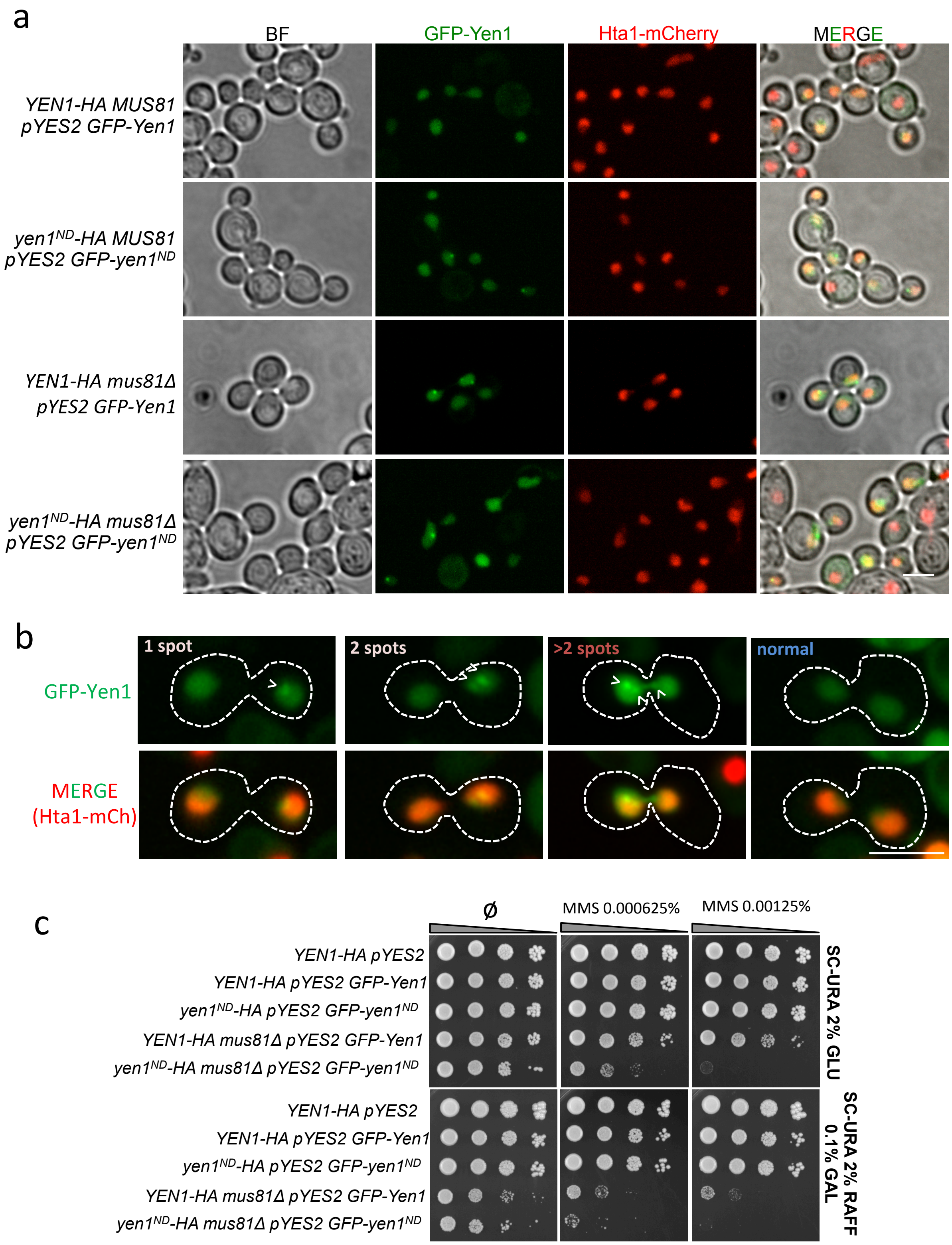

Supplementary Figure 2 a) Representative fields of strains expressing GFP-Yen1 on a short burst and its co-localizing signal with chromatin (Hta1) **b)** *slx8*Δ cells showing intra-nuclear foci that were categorized as 1-2 foci, >2 foci, normal or rare events (not included in the other categories) **c**) Strains used in microscopy were monitored for sensitivity to MMS under chronic low expression

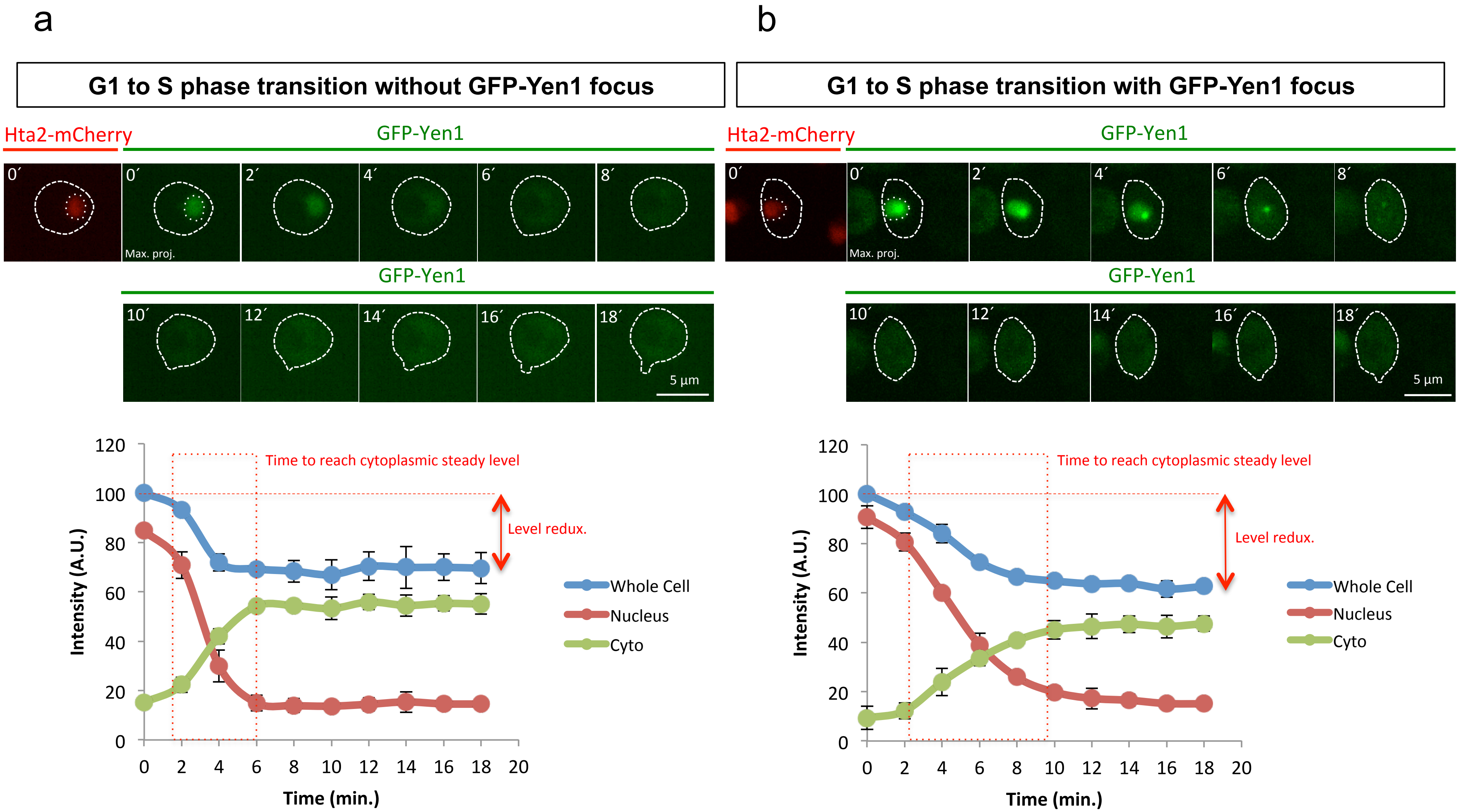

Supplementary Figure 3. **a)** Sequential images taken every 2 min of a cell expresing GFP-Yen1 during the re-location of GFP-Yen1 to the nucleus and associated graph showing the total intensity (normalized to 100%) of whole cell and both nuclear and cytoplasmic compartments for each time point. **b**) 2' time-lapse of a cell containing one focus during its GFP-Yen1 nuclear to cytoplasm re-distribution and associated graph (as in a). Graphs display the average intensity and SD (N=3).

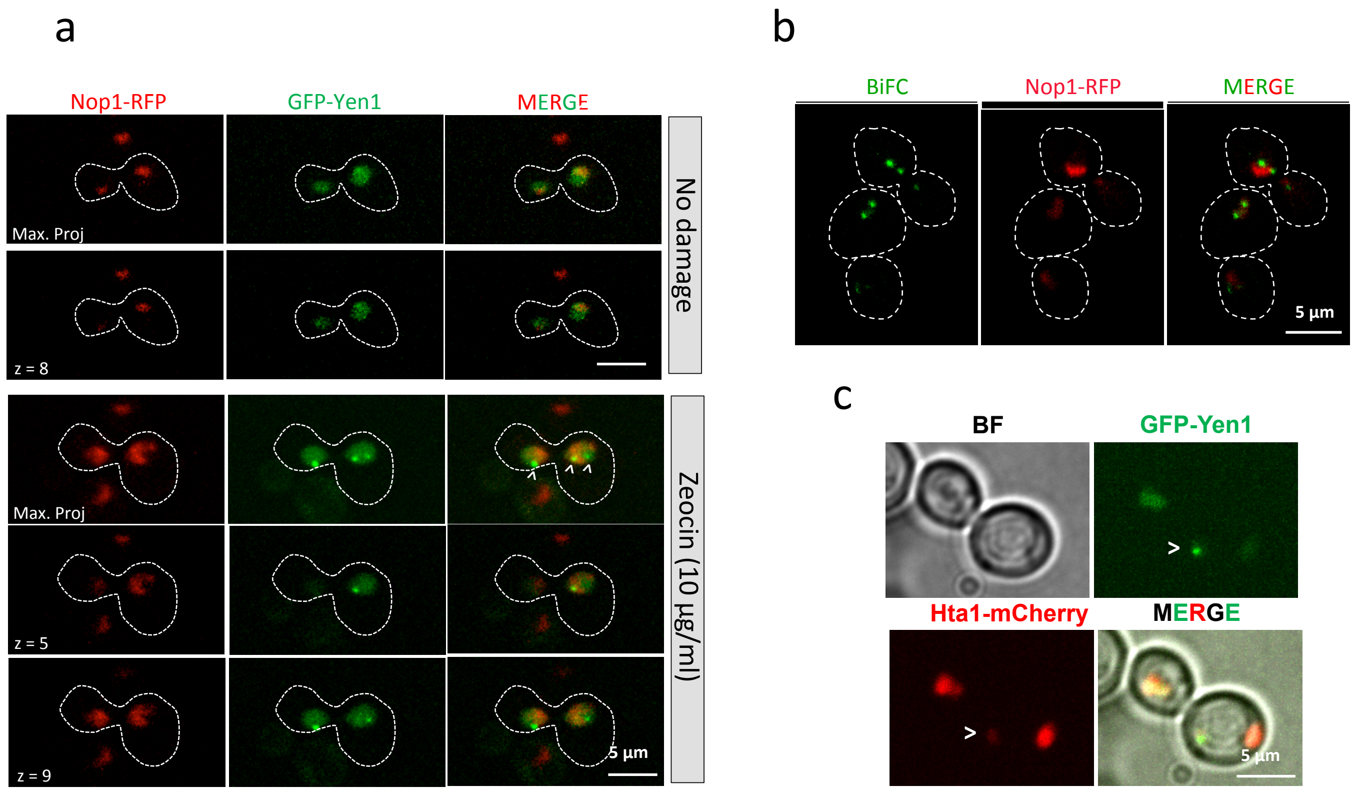

Supplementary Figure 4. **a)** Co-Localization of GFP-Yen1 and the Nop1-RFP nucleolar marker in normal and Zeocin challenge conditions. Co-localizing signal of Nop1 can also be detected with BiFC signal between Yen1 and Slx5. **b**) Co-localization of BiFC signal of the Slx5-Yen1 interaction with the Nop1 nucleolar marker. **c)** Representative image of a GFP-Yen1 focus localized to chromatin still not completely segregated in a *slx8*Δ cell (white arrow).

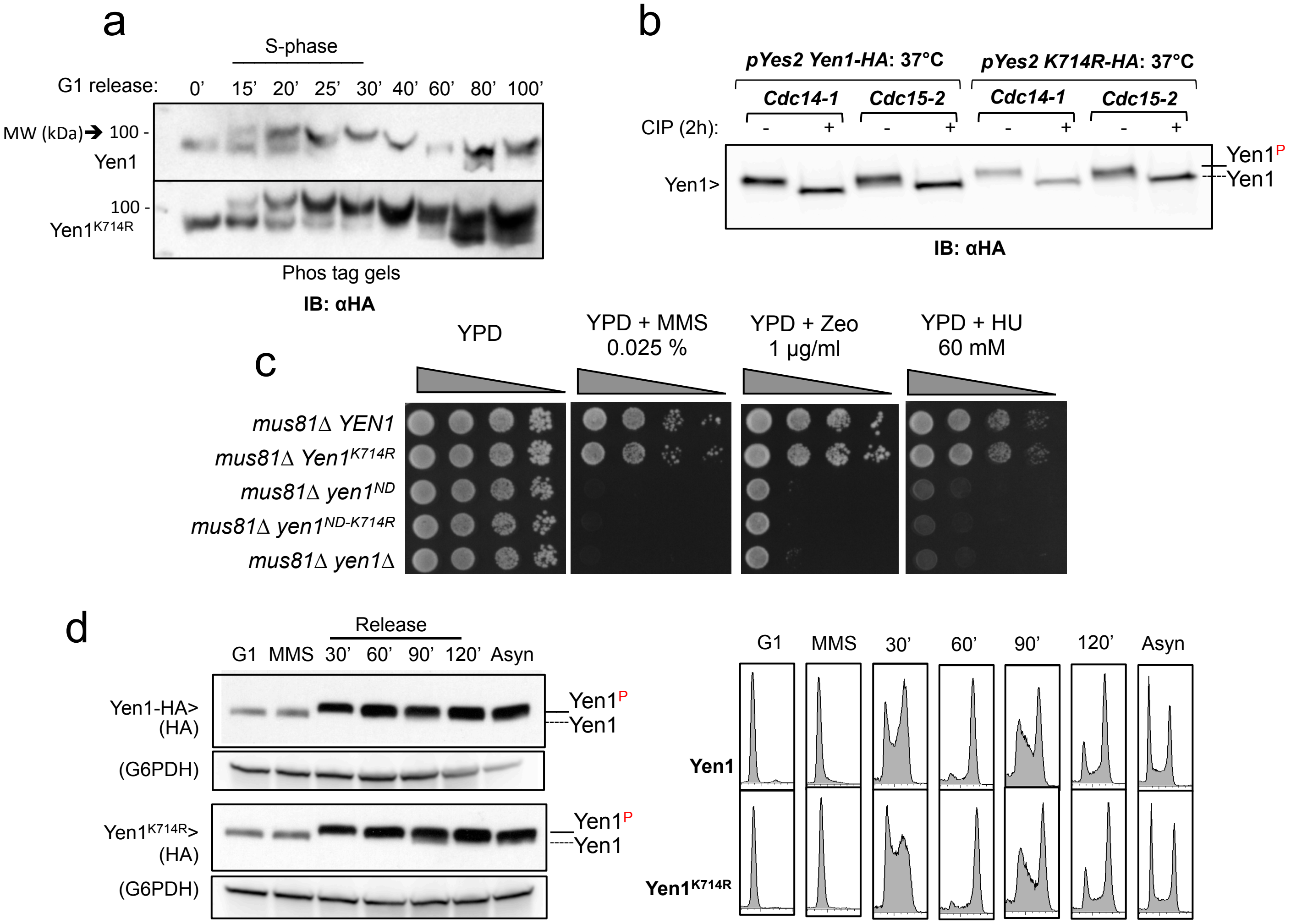

Supplementary Figure 5. a) PAGE phos-tag gel comparing phosphorylation status of a wild-type and a *yen1*-K714R strain during a time course after G1 release. **b**) *cdc14*-1 or *cdc15*-3 cells were arrested at restrictive temperature and protein extracts were either mock treated or treated with CIP phosphatase to reveal the extent of Cdc14- sensitive phosphorylation in both wild-type and *yen1*-K714R strains. **c**) Indicated strains (carrying a nuclease-dead ND allele of Yen1 combined or not with the K714R mutation) were subjected to a spot-test sensitivity assay by dropping serial dilutions in plates with the indicated genotoxics. **d**) Cells arrested in G1 were released in MMS (0.1%) containing media for 10' and washed out of drug and let recover in fresh YPD, samples at indicated points were analyzed by western-blot and flow-cytometry to monitor the MMS recovery.

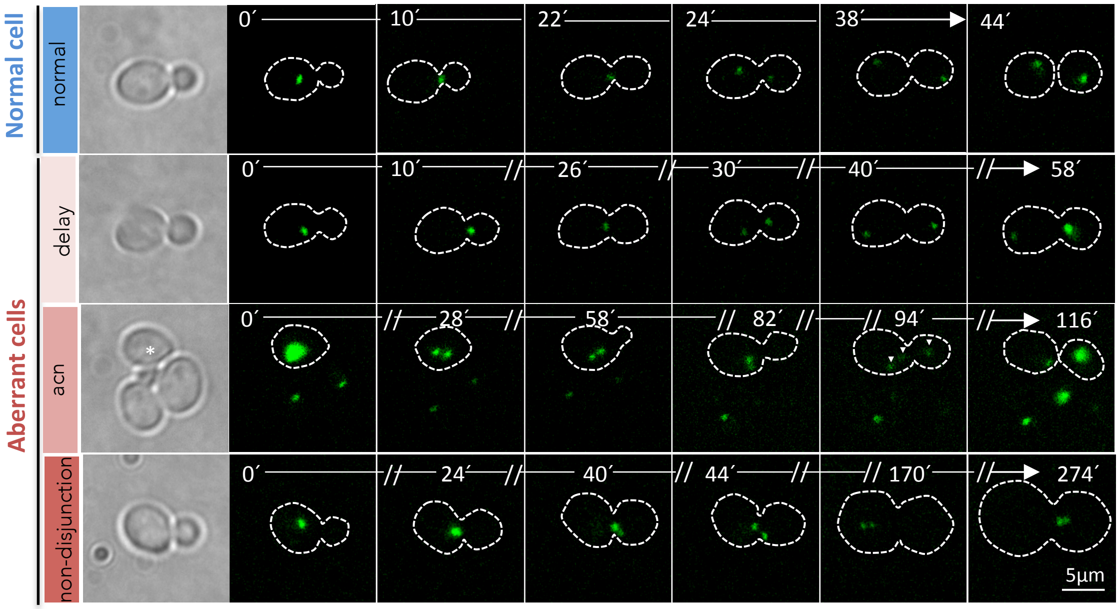

Supplementary Figure 6. Images of representative cells from the 4 different categories used to analyzed proper segregation by video-microscopy (using the LacI-GFP-LacO array). Note the time lapses are not equivalent and delayed and non-disjunction events have significant increases on the time between frames that illustrate its phenotype (acn, aberrant chromosome number).

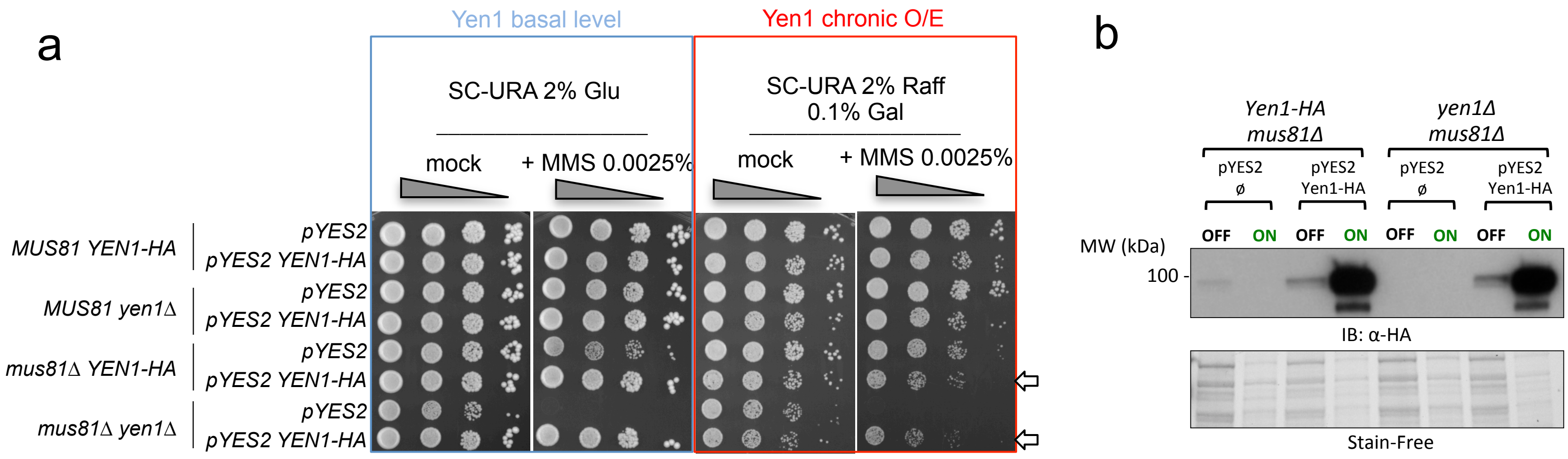

Suuplementary Figure 7. **a**) Strains carrying either a pYES2 empty vector or a pYES2-Yen1-HA expression vector were spotted in selective media with or without 0.0025% MMS in conditions allowing basal expression (Glucose repression) or chronic over-expression of YEN1 (Induction with Galactose). **b**) Analysis by western blot of the expression level of strains used in the segregation assay with acute O/E of Yen1-HA in Figure 7.

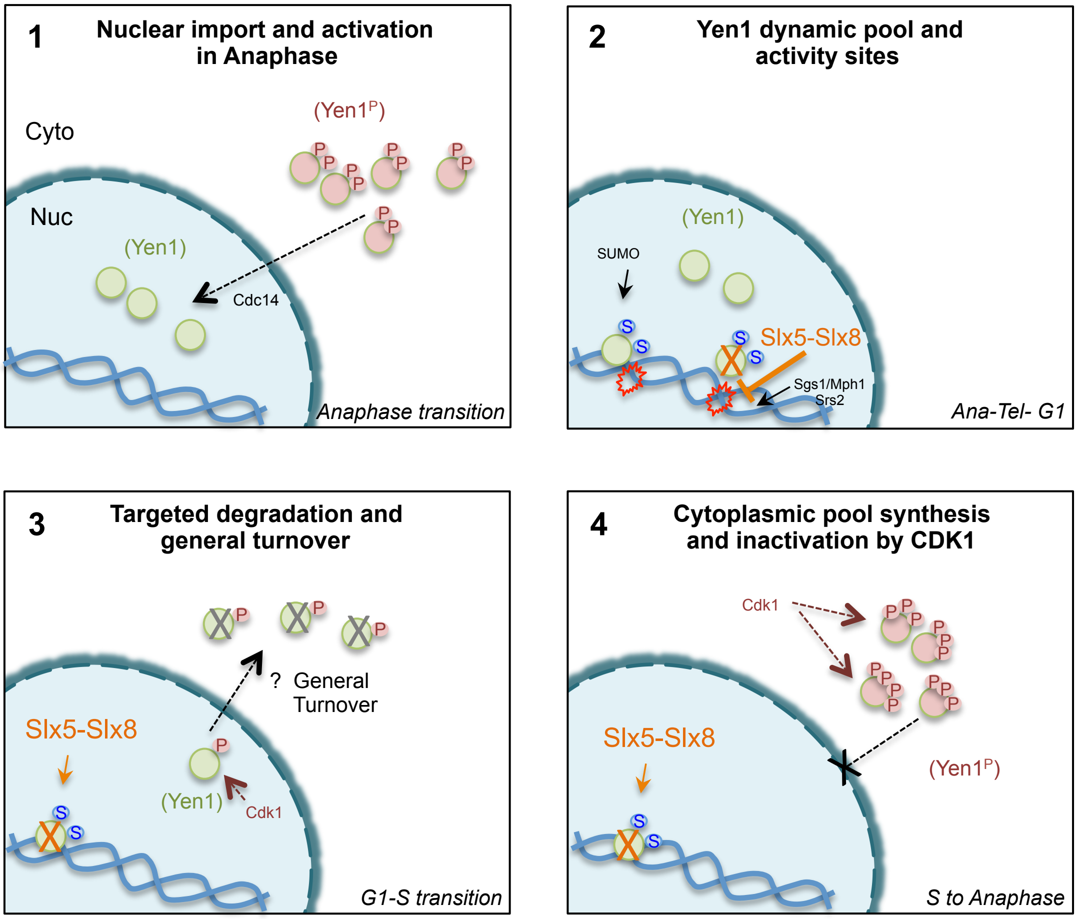

Supplementary Figure 8. **Model explaining the role of Slx5-Slx8 in targeting the active fraction of Yen1 for degradation**. At Anaphase transition (1), the cytoplasmic pool of Yen1 (pink circles) is still phosphorylated by Cdk1 and remains excluded from the nucleus. The action of Cdc14 enables active forms of Yen1 (green circles) to enter the nucleus and be recruited at its active sites with a putative role for sumoylation (2). Slx5-Slx8 removes Yen1 from active sites reducing the time of its association in competition with other HR factors (Sgs1, Mph1, Srs2) (2). After mitosis the Yen1 pool remains nuclear until Cdk1 gradually phosphorylates Yen1 at the entry of S-phase (3). During the G1-S transition the Yen1 pool is targeted to degradation in parallel to its nuclear exclusion (3) and the newly synthesized pool remains cytoplasmic after Cdk1 phosphorylation (4). Any Yen1 that remains in the nucleus is targeted by Slx5-Slx8 to allow its degradation and prevents its persistent accumulation in the nuceus at the S-phase (4).

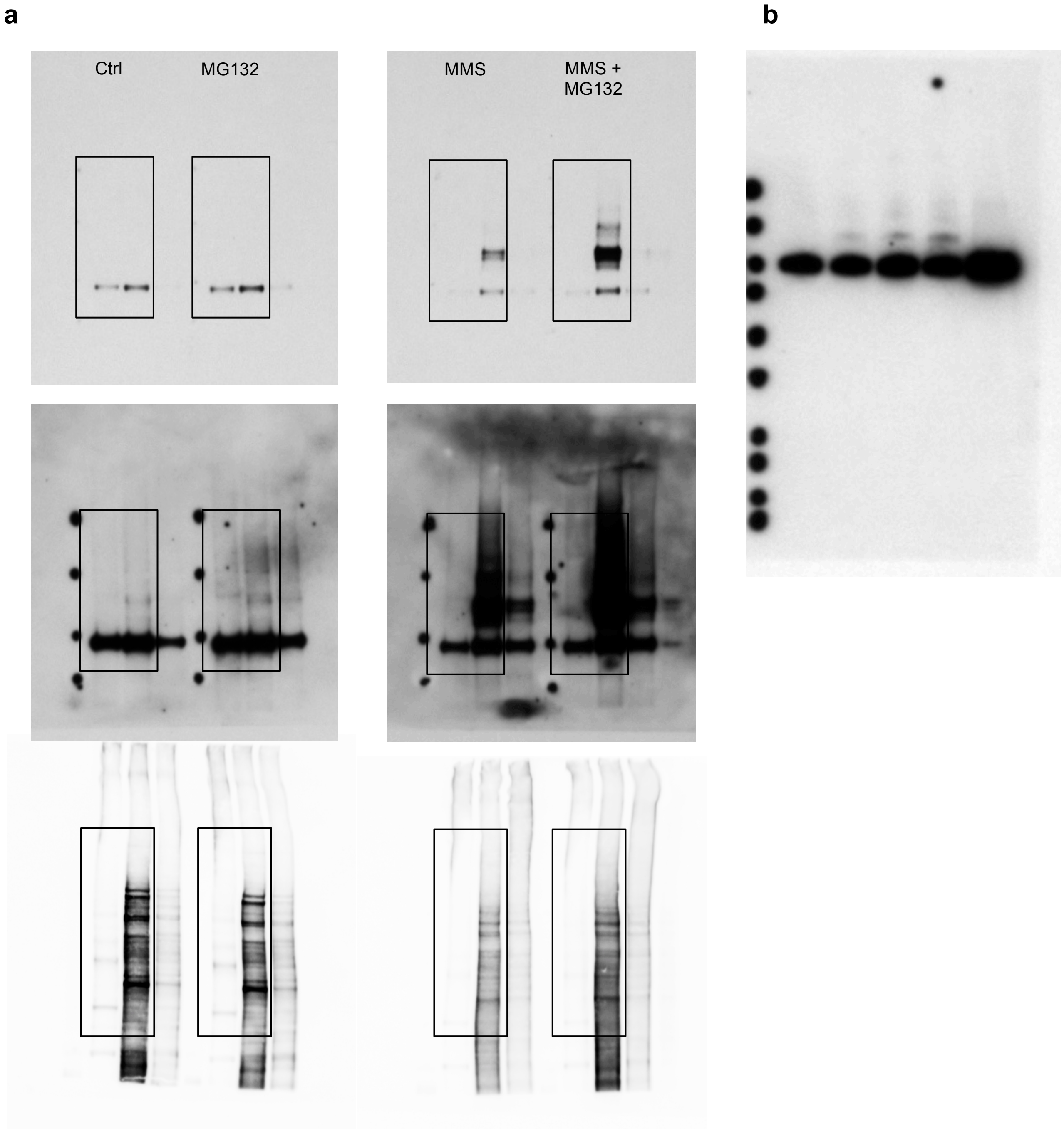

Supplementary Figure 9. a) Uncropped immunoblot images from Figure 1 panel b. Squares show the cropping limits used to generate the images in display in panel b. **b**) Uncropped blot used in Figure 1 panel e input control.

Supplementary Table 1. Yeast strains

Strain	Genotype*	Source or reference
**Strains for general purpose**		
GM77	*MAT**a** ADE2 ura3Δ::HphMX*	LSY2307-7B (Ho et al., 2010)
GM104	*MAT***a** ADE2 mus81Δ::KanMX ura3Δ::HphMX	LSY2307-1D (Ho et al., 2010)
GM58	*MAT***a** ADE2 yen1::HIS3 ura3Δ::HphMX	LSY2307-6D (Ho et al., 2010)
GM47	*MATα ADE2 yen1::HIS3 mus81Δ::KanMX*	LSY1801-1D (Ho et al., 2010)
GM84	*MAT**a** ADE2 yen1Δ::klURA3* ura3Δ::HphMX	This study
GM93-5D	*MATα ADE2 yen1Δ::klURA3 mus81Δ::KanMX*	This study
GM98-4B	*MAT**a** ADE2 YEN1-HA ura3Δ::HphMX*	This study
GM98-7B	*MATα ADE2 YEN1-HA mus81Δ::KanMX ura3Δ::HphMX*	This study
GM481-2A	*MAT**a** ADE2 YEN1-HA mus81Δ::KanMX ura3Δ::HphMX*	This study
GM230-9	*MAT**a** yen1-K714R-HA ura3Δ::HphMX*	This study
GM240-8B	*MAT**a** yen1-K714R-HA mus81Δ::KanMX ura3-1*	This study
GM398-8	MAT**a** yen1- S500A S507A S513A S583A -HA ura3Δ::HphMX	This study
GM569-4	MAT**a** yen1- S500D S507D S513D S583D -HA ura3Δ::HphMX ura3-1	This study
GM395-5C	*MAT**a** ADE2 yen1::HIS3 ura3-1*	This study
GM395-14C	*MAT**a** ADE2 yen1::HIS3 mus81Δ::KanMX ura3-1*	This study
GM481-3D	*MAT**a** ADE2 YEN1-HA slx8Δ::KanMX ura3Δ::HphMX*	This study
GM481-9B	*MAT**a** ADE2 YEN1-HA mus81Δ::KanMX slx8Δ::KanMX ura3Δ::HphMX*	This study
GM548-6A	*MAT**a** ADE2 yen1::HIS3 slx8Δ::KanMX ura3-1*	This study
GM399-5	*MAT**a** ADE2 yen1-E193A E195A-HA (ND) ura3Δ::HphMX*	This study
GM410-1B	*MAT**a** ADE2 yen1-E193A E195A-HA (ND) mus81Δ::KanMX ura3Δ::HphMX*	This study
GM424-1A	*MAT α ADE2 yen1-E193A E195A K714R-HA (K714R ND) ura3Δ::HphMX*	This study
GM424-4B	*MAT**a** ADE2 yen1-E193A E195A K714R-HA (K714R ND) mus81Δ::KanMX ura3Δ::HphMX*	This study
GM198-1B	*MAT**a** ADE2 YEN1-3xFLAG ura3Δ::HphMX*	This study
GM198-6D	*MAT**a** ADE2 YEN1-3xFLAG mus81Δ::KanMX ura3Δ::HphMX*	This study
GM563-3B	*MATα ADE2 YEN1-HA slx5Δ::NatMX*	This study
GM529	*MAT**a** ADE2 yen1::HIS3 pdr5Δ::HphMX*	This study

GM548-1B	*MAT***a** *ADE2 YEN1-HA pdr5Δ::HphMX*	This study
GM548-17A	*MAT***a** *ADE2 YEN1-HA slx8Δ::KanMX pdr5Δ::HphMX ura3-1*	This study
GM571-9B	*MAT***a** *ADE2 YEN1-HA slx5Δ::KanMX pdr5Δ::HphMX ura3-1*	This study
GM575	*MAT***a** *ADE2 yen1-K714R-HA pdr5Δ::HphMX ura3-1*	This study
GM635	*MAT***a** *ADE2 YEN1-HA siz1Δ::KanMX pdr5Δ::HphMX ura3-1*	This study
GM636	*MAT***a** *ADE2 YEN1-HA siz2Δ::KanMX pdr5Δ::HphMX ura3-1*	This study
GM672-9C	*MAT***a** *ADE2 YEN1-HA siz1Δ::KanMX siz2Δ::KanMX pdr5Δ::HphMX ura3-1*	This study
**Strains for cell biology**		
GM23	MATα his3-11:pCUP1-GFP12-LacI12:HIS3 trp1-1:256LacO:TRP1 lys2Δ bar1 ipl1-315-Flag:KanMX	SBY1372 (Kotwaliwale et al., 2007)
GM304-11C	*MATa* ADE2 Yen1-HA his3-11:pCUP1-GFP12-LacI12:HIS3 trp1-1:256LacO:TRP1 ura3Δ::HphMX	This study
GM371-10A	*MATa* ADE2 *yen1-K714R*-HA his3-11:pCUP1-GFP12-LacI12:HIS3 trp1-1:256LacO:TRP1 ura3Δ::HphMX	This study
GM304-6A	*MAT*α ADE2 Yen1-HA mus81::KanMX his3-11:pCUP1-GFP12-LacI12:HIS3 trp1-1:256LacO:TRP1 ura3Δ::HphMX	This study
GM249-2D	*MAT*α ADE2 *yen1-K714R*-HA mus81::KanMX his3-11:pCUP1-GFP12-LacI12:HIS3 trp1-1:256LacO:TRP1 ura3Δ::HphMX	This study
GM120-12A	*MAT*α ADE2 yen1::HIS3 mus81::KanMX his3-11:pCUP1-GFP12-LacI12:HIS3 trp1-1:256LacO:TRP1 ura3Δ::HphMX	This study
GM120-12B	MATα ADE2 yen1::HIS3 his3-11:pCUP1-GFP12-LacI12:HIS3 trp1-1:256LacO:TRP1 ura3Δ::HphMX	This study
GM524-2C (G)	*MATα ADE2 YEN1-HA hta1-mCHerry::HphMX (+pYES2-GFP-Yen1)*	This study
GM524-10A (G)	*MATα ADE2 YEN1-HA slx8Δ::KanMX hta1-mCherry::HphMX (+pYES2-GFP-Yen1)*	This study
GM564-1B (G)	*MATα ADE2 YEN1-HA slx5Δ::NatMX hta1-mCHerry::HphMX (+pYES2-GFP-Yen1)*	This study
GM374 (G)	*MATα ADE2 Yen1-K714R-HA hta1-mCherry::HphMX (+pYES2-GFP-yen1-K714R)*	This study
GM392 (G)	*MATα ADE2 YEN1-HA mus81Δ::KanMX hta1-mCherry::HphMX (+pYES2-GFP-Yen1)*	
GM556 (G)	*MAT***a** *ADE2 yen1-EE-HA hta1-mCherry::HphMX (+pYES2-GFP-yen1-EE)*	This study
GM525	MAT*α* ADE2 GFP-RAP1::LEU2 SIK1-mRFP::KanMX	yKD939 (Batte et al., 2017)
GM531-1B (G)	MATα ADE2 *YEN1*-HA SIK1-mRFP::KanMX (+ pYES2-GFP-Yen1)	This study
GM560-12D (G)	MAT**a** ADE2 *YEN1*-HA *SIK1*-mRFP::KanMX *slx8Δ*::KanMX (+ pYES2-GFP-Yen1)	This study
**Strains for crossover monitoring**		
LSY2205-24D	*MATα ade2-I lys2::GAL-ISCEI his3::HphMX4 yen1::HIS3*	(Ho et al., 2010)

LSY2202-42A	*MAT***a** *ade2-n his3::NatMX4 met22::klURA3 yen1::HIS3*	(Ho et al., 2010)
LSY2205-77B	*MAT***a** *ade2-I lys2::GAL-ISCEI his3::HphMX4 mus81::KanMX6 yen1::HIS3*	(Ho et al., 2010)
LSY2202-19D	*MATα ade2-n his3::NatMX4 met22::klURA3 mus81::KanMX6 yen1::HIS3*	(Ho et al., 2010)
GM379-4C	MATa YEN1-HA ade2-I lys2Δ::pGal-ISceI his3Δ::HphMX	This study
GM379-13C	*MATα YEN1-HA mus81Δ::KanMX ade2-I lys2::GAL-ISCEI his3::HphMX4*	This study
GM387-22B	*MATα YEN1-HA ade2-n his3::NatMX4 met22::klURA3*	This study
GM387-5A	*MAT***a** *YEN1-HA mus81Δ::KanMX ade2-n his3::NatMX4 met22::klURA3*	This study
GM244-1B	*MAT***a** *yen1-K714R-HA ade2-I lys2::GAL-ISCEI his3::HphMX4*	This study
GM243-9A	*MATα yen1-K714R-HA ade2-n his3::NatMX4 met22::klURA3*	This study
GM243-3B	*MAT***a** *yen1-K714R-HA mus81Δ::KanMX ade2-n his3::NatMX4 met22::klURA3*	This study
GM244-7A	*MATα yen1-K714R-HA mus81Δ::KanMX ade2-I lys2::GAL-ISCEI his3::HphMX4*	This study
**Strains for BiFC (BY4741 and BY4742 backgrounds)**		
GM540	MATa KanMX::pGAL::VN-Yen1 his3Δ1 leu2Δ0 met15Δ0 ura3Δ0	This study
GM541	MAT*α* KanMX::pGAL::VC-Slx5 his3Δ1 leu2Δ0 lys2Δ0 ura3Δ0	This study
**Strains for Two-Hybrid**		
PJ69-4a	*MAT***a** trp1-901leu2-3.12 ura3-52 his3-200 gal4Δ gal80Δ LYS2::GAL1-HIS3 GAL2-ADE2 met2::GAL7-lacz	Stan Fields Lab (James et al., 1996)
Pj69-4alpha	*MATα* trp1-901leu2-3.12 ura3-52 his3-200 gal4Δ gal80Δ LYS2::GAL1-HIS3 GAL2-ADE2 met2::GAL7-lacz	Stan Fields Lab (James et al., 1996)
**Construction with tetO-chr XII**		
LSY2282-1B	MATa ADE2 ura3:3xURA3 - tetO x112, TetR-mRFP yen1::HIS3	derived from W6956-24D (Rodney Rothstein Lab)
GM109-15C	MATα Yen1-HA mus81Δ::KanMX ura3:3xURA3-tetOx112 TetR-mRFP	This Study
GM518	MATa his3-Δ1, leu2-Δ1, ura3-Δ0 ade2-801 lys2-801, LYS2::TETR-GFP, nup49Δ::HphMX, inter YLR188w-YLR189c::ura3::TetO-NATMX (+ pASZ11-NupNop)	HBT28_1a (Albert et al., 2013)
GM562-5C	MAT*α* *ADE2 YEN1-HA* inter YLR188w-YLR189c::ura3::TetO-NatMX TetR-RFP *ura3*Δ::HphMX met17s his3?, leu2? (+ pYES2-GFPYen1)	Cross of HBT28_1a with 109-15C and 2 re-cross on 109-15C

*If not stated otherwise strains background is the W303 genotype (*his3-11, 15 leu2-3, 112 trp1-1 ade2-1 can1-100*), only mating type and differences from the standard genotype are listed. Specific strains in other backgrounds are defined.

Supplementary Table 2. Plasmids

Plasmid	Description
pNJ7766-Yen1	*pET21a derivative encoding 6xHIS-HA-Yen1 (IPTG inducible)*
pNJ7766-yen1-K714R	*pET21a derivative encoding 6xHIS-HA-yen1-K714R(IPTG inducible)*
pYES2	*pYES2 (URA3) empty vector, GAL inducible*
pYES2-Yen1-HA	*pYES2 (URA3) derivative, GAL inducible expressing wild type Yen1-HA*
pYES2-yen1-K714R-HA	*pYES2 (URA3) derivative, GAL inducible expressing yen1-K714R-HA*
pYES2-Yen1-3xFLAG	*pYES2 (URA3) derivative, GAL inducible*
pYES2-GFP-Yen1	*pYES2-TOPO (URA3) derivative, GAL inducible*
pYES2-GFP-Yen1$_{ND}$	*pYES2-TOPO (URA3) derivative, GAL inducible*
pYES2-GFP-yen1-K714R	*pYES2-TOPO (URA3) derivative, GAL inducible*
pRS315	*empty vector (LEU2)*
p1346	*Cu inducible (LEU2) encoding 6xHIS-Smt3 (from B. Palancade)*
pJM421	*Cu inducible (LEU2) encoding Ubi4*
pJD421	*Cu inducible (LEU2) encoding 6xHIS-Ubi4*
pOA-Slx5	*AD fusion with Slx5 (TRP1), derived from pOAD (from Stan Fields)*
pBDB-Yen1	*DBD fusion with Yen1 (LEU2), derived from pOBD2 (from Stan Fields)*
pUN100-mCherry-NOP1	*pRS305 derivative (LEU2) encoding mCherry-NOP1 (constitutive) (from O. Gadal)*
p1028-NUP49-mCherry (LEU2)	*pRS305 derivative (LEU2) encoding mCherry-Nup49 (from B.Palancade)*
p1069 His-Flag-Smt3 (LEU2)	Gal inducible (LEU2) encoding His-Flag-Smt3
pYES2 GST-Slx5	*pYES2-TOPO (URA3) derivative, GAL inducible expressing the fusion protein GST-Slx5*

Supplementary methods

Yeast Strains and Plasmids

S. cerevisiae strains used in this study are listed in Table S1. Strains were generated by crossing and are derivatives of the W303c background. The Yen1-HA allele was generated by inserting a FactorXa cleavage site and a single -HA epitope at its C-terminus using PCR amplification with a dedicated oligonucleotide. Full length YEN1-HA was transformed into a *yen1*Δ::KlURA3 strain followed by counter-selection on 5-FOA to insert the allele in the endogenous locus. Positive clones were confirmed by sequencing and back-crossed to a wild-type W303c strain. The pYES2-GFP-Yen1 plasmid was obtained by cloning the GFP-Yen1 allele from pGAD-GFP-Yen1 [63] into pYES2-TOPO2 vector. Vectors expressing Yen1-HA or Yen1-3xFLAG were generated by inserting a PCR fragment of Yen1 with the tag and restriction sites into pYES2 opened by *HindIII* and *NotI*. Mutations in Yen1 were generated by PCR mutagenesis and introduced into the endogenous locus by transformation and gene replacement with a full-length YEN1 ORF PCR product into a *yen1*Δ::KlURA3 strain. Mutants in the different designated loci where either obtained by crossing or by gene replacement with the designated selective cassettes. Plasmid pNJ7766 contains the 6xHIS-1xHA-Yen1 insert in pET21a for expression of N-terminal-tagged Yen1 in *E.coli*.

Smt3 and Ubi4 denaturing pull-downs

Strains containing the 1346 plasmid (pCUP1-6xHIS-Smt3) or the control plasmid were grown in SC-LEU modified medium (0.1% proline, 0.017% YNB without ammonium sulfate), allowing direct MG132 treatment. Overnight cultures were diluted in 100 ml to an OD600=0.2 in the same medium but containing 0.003% SDS. Cells were allowed to grow to OD600=0.3 when $CuSO_4$ was added at 100 μM final concentration. After 1hr MMS was added to 0.3% and cells were collected 3hrs later. For cultures inhibited for proteasome degradation MG132 was added to 100 μM 2 hrs before harvesting the cells. Cells where lysed under denaturing conditions and SUMO or

ubiquitin-conjugated proteins where isolated as described [39,66,67] with the following modifications. Cells were precipitated 30 min on ice in 10% TCA, and lysed with glass beads for 20 min at 4°C. After centrifugation, the pellet was washed with cold acetone, and then we resuspended the pellet in guanidine buffer containing 6 M guanidium-HCl, 20 mM Tris-HCl (pH 8.0), 100 mM Na_2HPO_4, 10 mM imidazole, 100 mM NaCl, 0.1% Triton X-100, 0.05% Tween-20, 10 mM β-mercaptoethanol, 100 µM MG132 and 50 mM NEM (N-Etyl-Malemeide) and incubated the sample 1 hour at room temperature on rotating platform. After centrifugation at room temperature during 10 min at 16,000 g, the lysate was incubated for 2.5 hours with agarose nickel-nitriloacetic-acid beads (Agarose Ni-NTA ; Qiagen) previously equilibrated with guanidine buffer. The matrix was then washed three times with Guanidine buffer and three times with urea buffer containing 8 M urea, 100 mM Na_2HPO_4 (pH 6.3), 10 mM Tris-HCl (pH 8.0), 0.1% Triton X-100, 10 mM β-mercaptoethanol and 100 µM MG132. Samples were routinely treated with CIP phosphatase to allow a clearer detection of sumoylation and ubiquitination. Beads were dried before proceeding to elution in HU buffer composed by 8 M urea, 200 mM Tris-HCl (pH 6.8), 1 mM EDTA, 5% SDS, 0.1% bromophenol blue and 1.5 % DTT. Samples were heated at 65°C for 20 min. Eluates were loaded in 3-8% NuPAGE Tris-Acetate gradient gels. After transfer, membranes were processed for immuno-detection and finally stained with Ponceau Red to confirm sample loading. Input samples served as control in standard SDS-PAGE gels for equivalent expression of Yen1-HA.

DSB-induced *ade2* recombination assay

Diploid strains containing 2 hetero-alleles of ade2 that are cleaved by I-SceI and repaired to give rise to either ADE2 or ade2-n repair products in three types of colonies (red, white and sectored) were grown in YP-R (2%) Overnight. Cultures were diluted and let grow to exponential phase when the DSB was induced by addition ogf Galactose at 2%. Cells were immediately plated in YP-D (2%) and grown for 3 days.

Colonies were scored as solid red, solid white and sectored and replica plated to YP-D containing Hygromycin (SIGMA), Nourseothricin (Werner BioAgents), both antibiotics and to SC-URA-MET and SC-ADE (Raffinose 2% Galactose 2%). Non-recombinants were detected and excluded from analysis by papillation growth in YP-D and growth as ADE2 reversion in SC-Ade+Raff+Gal. Outcomes were scored by assigning to each colony the recombination events that correspond to the repair of each of the two sister-chromatids. A colony retaining heterozygosity for *Nat* and *Hph* was scored as two NCO events while a sectored colony with reciprocal LOH was scored as one NCO and one CO event. Recombination outcomes are presented relative to the YP-Gal (galactose) versus YP-D (glucose) PE of the strains (mean ± SD). To better address PE, cells were deposited by micromanipulation in both media plates and colony growth was scored after 3 days. Statistical significance was determined by Fisher's exact test between two categories with the indicated N (number of chromatid outcomes) shown in the legend. All strains were induced independently at least three times (see number of trials) and the results of each induction were pooled to calculate the distribution of events.

References

Albert, B., Mathon, J., Shukla, A., Saad, H., Normand, C., Leger-Silvestre, I., Villa, D., Kamgoue, A., Mozziconacci, J., Wong, H., *et al.* (2013). Systematic characterization of the conformation and dynamics of budding yeast chromosome XII. J Cell Biol *202*, 201-210.

Batte, A., Brocas, C., Bordelet, H., Hocher, A., Ruault, M., Adjiri, A., Taddei, A., and Dubrana, K. (2017). Recombination at subtelomeres is regulated by physical distance, double-strand break resection and chromatin status. EMBO J *36*, 2609-2625.

Ho, C.K., Mazon, G., Lam, A.F., and Symington, L.S. (2010). Mus81 and Yen1 promote reciprocal exchange during mitotic recombination to maintain genome integrity in budding yeast. Mol Cell *40*, 988-1000.

James, P., Halladay, J., and Craig, E.A. (1996). Genomic libraries and a host strain designed for highly efficient two-hybrid selection in yeast. Genetics *144*, 1425-1436.

Kotwaliwale, C.V., Frei, S.B., Stern, B.M., and Biggins, S. (2007). A pathway containing the Ipl1/aurora protein kinase and the spindle midzone protein Ase1 regulates yeast spindle assembly. Dev Cell *13*, 433-445.
